# The m^6^A reader IGF2BP2 regulates glycolytic metabolism and mediates histone lactylation to enhance hepatic stellate cell activation and liver fibrosis

**DOI:** 10.1038/s41419-024-06509-9

**Published:** 2024-03-05

**Authors:** Yongqiang Zhou, Jiexi Yan, He Huang, Lu Liu, Longfei Ren, Jinjing Hu, Xiaoxu Jiang, Yan Zheng, Lingcong Xu, Fupeng Zhong, Xun Li

**Affiliations:** 1 The First School of Clinical Medicine, Lanzhou University, Lanzhou, China; 2 Precision Medicine Center, The First Hospital of Lanzhou University, Lanzhou, China; 3 Department of Pediatrics, The First Hospital of Lanzhou University, Lanzhou, China; 4 Department of General Surgery, The First Hospital of Lanzhou University, Lanzhou, China; 5 Key Laboratory of Biotherapy and Regenerative Medicine of Gansu Province, Lanzhou, China

**Keywords:** RNA modification, Post-translational modifications

## Abstract

Evidence for the involvement of N^6^-Methyladenosine (m^6^A) modification in the etiology and progression of liver fibrosis has emerged and holds promise as a therapeutic target. Insulin-like growth factor 2 (IGF2) mRNA-binding protein 2 (IGF2BP2) is a newly identified m^6^A-binding protein that functions to enhance mRNA stability and translation. However, its role as an m^6^A-binding protein in liver fibrosis remains elusive. Here, we observed that IGF2BP2 is highly expressed in liver fibrosis and activated hepatic stellate cells (HSCs), and inhibition of IGF2BP2 protects against HSCs activation and liver fibrogenesis. Mechanistically, as an m^6^A-binding protein, IGF2BP2 regulates the expression of Aldolase A (*ALDOA*), a key target in the glycolytic metabolic pathway, which in turn regulates HSCs activation. Furthermore, we observed that active glycolytic metabolism in activated HSCs generates large amounts of lactate as a substrate for histone lactylation. Importantly, histone lactylation transforms the activation phenotype of HSCs. In conclusion, our findings reveal the essential role of IGF2BP2 in liver fibrosis by regulating glycolytic metabolism and highlight the potential of targeting IGF2BP2 as a therapeutic for liver fibrosis.

## Introduction

Liver fibrosis is a prevalent pathological process observed in various chronic liver diseases, characterized by the excessive deposition of extracellular matrix (ECM) [1]. Apart from removal of the underlying cause or liver transplantation, no effective treatment for liver fibrosis has yet been identified [2]. The activation and phenotypic transformation of quiescent hepatic stellate cells (HSCs) are believed to play a crucial role in initiating liver fibrosis [3]. This activation process necessitates metabolic reprogramming, including the adoption of aerobic glycolysis and increased energy availability [4]. Aerobic glycolysis serves as a significant metabolic signature of HSCs activation [5]. A growing body of evidence suggests that HSCs employ aerobic glycolysis during activation, and inhibiting this process effectively halts HSCs activation [6–8]. However, the precise role and mechanism of lactate accumulation resulting from aerobic glycolysis in the activation of HSCs remain unclear.

Lactate has traditionally been regarded as a byproduct of anaerobic glycolysis with no significant biological function. However, emerging evidence suggests that lactate can serve as a crucial bio-signaling molecule involved in immunomodulation and gene expression, in addition to its role as an energy source [9]. Recent studies have identified lactate as a substrate for histone lactylation, a process in which lactate accumulated during cellular metabolism modifies lysine residues on histones, thereby promoting the transcription of specific genes [10]. Notably, histone lactylation has been found to impact tumorigenesis [11–13], endothelium function [14], and neuromodulation [15]. Consequently, the study of histone lactylation holds great potential for advancing our understanding of the underlying mechanisms of liver fibrosis.

Insulin-like growth factor 2 (IGF2) mRNA-binding protein 2 (IGF2BP2) is a member of the IGF2 mRNA-binding protein family (IGF2BPs) and functions as an RNA-binding protein that governs diverse biological processes. It possesses several RNA-binding structural domains, namely four C-terminal heterologous ribonucleoprotein K homology (KH) (KH1-KH4) domains and two N-terminal RNA recognition motifs [16], and are involved in the regulation of a wide range of RNA processing mechanisms, such as localization, stability, and translation [17]. Accumulating evidence suggests that IGF2BP2 plays a significant role in various liver diseases, such as nonalcoholic steatohepatitis [18], fatty liver [19], hepatic steatosis [20], liver fibrosis [21], and liver cirrhosis [22]. It is worth noting that there is an increasing body of evidence indicating that IGF2BP2 regulates metabolic pathways in cells through post-transcriptional mechanisms, including glycolysis [23, 24], glutamine metabolism [25], and lipid metabolism [20]. However, the impact of IGF2BP2-mediated metabolic changes on liver fibrosis remains uncertain.

In this study, we present evidence suggesting that IGF2BP2 plays a role in liver fibrogenesis by regulating glycolytic metabolism. Our findings indicate that IGF2BP2 is upregulated in liver fibrosis, and silencing IGF2BP2 protects against carbon tetrachloride (CCl_4_)-induced liver fibrosis. In vitro experiments further demonstrate that silencing IGF2BP2 inhibits the activation of HSCs. Mechanistically, we propose that IGF2BP2 enhances the stability of the m^6^A-modified transcript Aldolase A (*ALDOA*) in the glycolytic metabolic pathway, which is crucial for HSC activation. Additionally, our study reveals that lactate produced by activated HSCs acts as a substrate for lactylation, and correcting aberrant lactylation level can alter the activation phenotype of HSCs.

## Results

### IGF2BP2 expression is elevated in liver fibrosis and cirrhosis

**Fig. 1:**
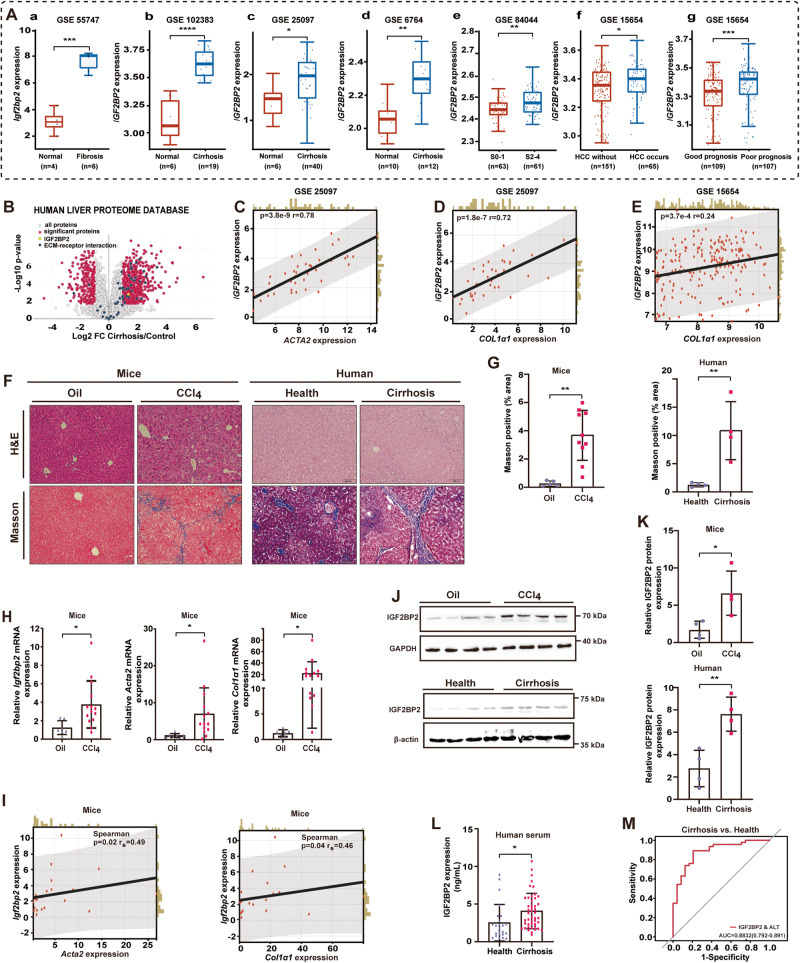
IGF2BP2 expression is elevated in liver fibrosis and cirrhosis. **A** *IGF2BP2* expression in liver fibrosis and cirrhosis from the GEO database. (**a**) *Igf2bp2* expression in CCl_4_-induced liver fibrosis and normal control from GSE55747. **b**–**d** *IGF2BP2* expression in liver cirrhosis and normal control from GSE102383, GSE25097, and GSE6764. **e** *IGF2BP2* expression in patients with Scheuer scores S0-1 and S2-4 from GSE84044. **f** *IGF2BP2* expression in cirrhotic patients who develop HCC and those who do not develop HCC from GSE15654. **g** *IGF2BP2* expression in cirrhotic patients with good or poor prognosis from GSE15654. **B** IGF2BP2 expression in cirrhosis retrieved from the Human Liver Proteome Database. **C**–**E** Correlation analysis of *IGF2BP2* expression with *ACTA2* and *COL1α1* expression in cirrhotic samples from GSE25097 (*n* = 40) and GSE15654 (*n* = 216). Representative H&E staining and Masson’s trichrome staining images (**F**) and bar plot (**G**) in CCl_4_-induced fibrotic livers of mice and livers of human patients with cirrhosis (scale bars, 100 µm). **H** *Igf2bp2*, *Acta2*, and *Col1α1* mRNA expression in CCl_4_-induced fibrotic livers of mice. **I** Correlation analysis of *Igf2bp2* mRNA expression with *Acta2* mRNA expression and *Col1α1* mRNA expression. Levels of IGF2BP2 in CCl_4_-induced fibrotic livers of mice and livers of human patients with cirrhosis revealed by immunoblotting (**J**) and bar plot (**K**). **L** Serum levels of IGF2BP2 in cirrhotic patients (*n* = 46) and healthy donors (*n* = 24). **M** The receiver operating characteristic (ROC) curve demonstrates the efficacy of IGF2BP2 combined with alanine transaminase (ALT) in predicting cirrhosis. *P < 0.05, **P < 0.01, ***P < 0.001, ****P < 0.0001.

Initially, we evaluated the expression of *IGF2BP2* by examining multiple Gene Expression Omnibus (GEO) datasets. Through the analysis of the GSE55747 dataset, we observed an increased expression of *Igf2bp2* in liver fibrosis induced by CCl_4_ (Fig. 1a). In concurrence with the findings from the GSE102383, GSE25097, and GSE6764 datasets, it has been observed that the expression of *IGF2BP2* is significantly increased in cirrhotic livers when compared to normal control livers (Fig. 1b–d). Moreover, according to the GSE84044 dataset, it was observed that the expression of *IGF2BP2* was increased in individuals with notable fibrosis (scheuer score S2-4) in comparison to those without significant fibrosis (scheuer score S0-1, Fig. 1e). In a follow-up dataset analysis involving individuals diagnosed with cirrhosis (GSE15654), it was observed that patients with hepatocellular carcinoma (HCC) exhibited significantly higher levels of *IGF2BP2* expression compared to those without HCC (Fig. 1f). Furthermore, heightened *IGF2BP2* expression was found to be associated with a poorer prognosis in these patients (Fig. 1g).

In the Human Liver Proteome Database (http://www.liverproteome.org/), IGF2BP2 was retrieved as a significantly expressed protein in liver cirrhosis (Fig. 1B). In a correlation study between *IGF2BP2* and representative markers of liver fibrosis, based on cirrhotic samples (*n* = 40) from the GSE25097 dataset, *ACTA2* (actin alpha 2) expression and collagen alpha 1 (*COL1α1*) expression both exhibited a substantial positive correlation with *IGF2BP2* expression (Fig. 1C, D. *p* = 3.8e-9, *r* = 0.78; *p* = 1.8e-7, *r* = 0.72, respectively). The dataset GSE15654 also demonstrated a positive correlation between the expression of *IGF2BP2* and *COL1α1* in cirrhotic samples (*n* = 216) (*p* = 3.7e-4, *r* = 0.24, Fig. 1E).

Subsequently, we employed CCl_4_ to induce liver fibrosis in mice, with the aim of investigating the expression of *IGF2BP2* in fibrotic liver tissues. Furthermore, we obtained samples of human livers, including both normal and cirrhotic liver tissues (*n* = 4 for each group). The liver tissues exhibited abnormal morphology and the presence of fibrosis, as evidenced by hematoxylin and eosin (H&E) staining and Masson’s trichrome staining (Fig. 1F, G) Significantly, the upregulation of *IGF2BP2* in fibrotic livers was validated through quantitative reverse transcription polymerase chain reaction (qRT-PCR) analysis, and demonstrated a parallel increase in the expression of key genes (*ACTA2*, *COL1α1*) associated with the advancement of liver fibrosis (Fig. 1H, I).

Immunoblotting analysis showed elevated levels of IGF2BP2 in fibrotic liver samples compared to normal liver tissue (Fig. 1J, K). These findings collectively suggest heightened expression of IGF2BP2 in fibrotic livers and provide substantial evidence for a significant correlation with human liver fibrosis.

In order to assess the potential utility of IGF2BP2 as a marker for cirrhosis, a total of 46 serum samples from cirrhotic patients and 24 samples from healthy individuals were collected. Enzyme linked immunosorbent assay (ELISA) revealed that patients with cirrhosis exhibited higher levels of IGF2BP2 in their serum (Fig. 1L). Encouragingly, the combination of IGF2BP2 and alanine aminotransferase (ALT) demonstrated efficacy in predicting the presence of liver cirrhosis, as determined by receiver operating characteristic (ROC) analysis (Fig. 1M).

### Inhibition of IGF2BP2 ameliorates CCl_4_-induced liver fibrosis

**Fig. 2:**
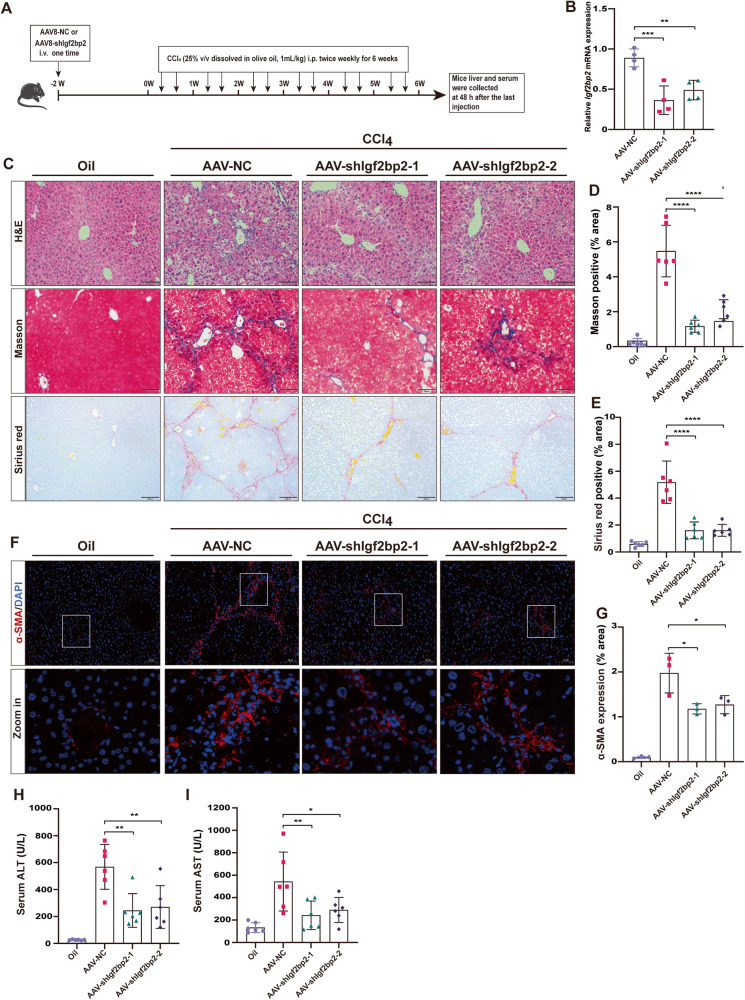
Inhibition of IGF2BP2 ameliorates CCl_4_-induced liver fibrosis. **A** Schematic illustrating of the experimental design of AAV-shIgf2bp2 for the treatment of CCl_4_-induced liver fibrosis in mice. **B** Expression of *Igf2bp2* in mouse liver samples treated in according to (**A**) (*n* = 4). Representative images (**C**) and bar plot (**D**, **E**) of H&E staining (upper), masson’s trichrome staining (middle) and sirius red staining (bottom) in mouse liver sections treated according to (**A**) (*n* = 6, scale bar, 100 µm). Representative images (**F**) and bar plot (**G**) of α-SMA expression (*n* = 3, scale bar, 100 µm) visualized by immunofluorescence in mouse liver sections treated according to (**A**). Serum levels of ALT (**H**) and AST (**I**) in mice treated according to (**A**). *P < 0.05, **P < 0.01, ***P < 0.001, ****P < 0.0001.

To confirm the role of IGF2BP2 in the development of liver fibrosis in vivo, we inhibited *Igf2bp2* expression by the adeno-associated virus (AAV)8 system, known for its strong affinity for the liver. Additionally, we simulated liver fibrosis induced by CCl_4_ and administered AAV8 treatment two weeks prior to the initial injection (Fig. 2A). The expression of *Igf2bp2* was notably hindered in the livers of mice following the AAV8 administration, indicating a successful infection with AA8-shIgf2bp2 (Fig. 2B). We subsequently investigated the incidence of fibrosis following AAV8 administration in the context of prolonged liver injury. Through the utilization of H&E staining, Masson’s trichrome staining, and sirius red staining, we observed that the severity of liver injury and fibrosis was reduced in mice treated with AAV-shIgf2bp2 compared with mice treated with AAV-NC (Fig. 2C–E). Immunofluorescence analysis consistently demonstrated that AAV8-shIgf2bp2 infection successfully inhibited the expression of α-smooth muscle actin (α-SMA) in the liver tissue of mice (Fig. 2F, G). Furthermore, the infection with AAV8-shIgf2bp2 exhibited a beneficial effect on liver function in mice, as evidenced by the reduction in serum levels of ALT and aspartate aminotransferase (AST) (Fig. 2H, I). In conclusion, the results of this study suggest that the suppression of IGF2BP2 expression in the liver can mitigate the advancement of liver fibrosis. Therefore, targeting IGF2BP2 may hold promise as a potential therapeutic strategy for the treatment of liver fibrosis.

### IGF2BP2 is upregulated in activated HSCs

The preceding data indicates IGF2BP2 potential involvement in the development of liver fibrosis. Given the significant involvement of HSCs activation in the development of liver fibrosis, our subsequent inquiry focused on examining the expression profile of IGF2BP2 in activated HSCs and its impact on the activation of these cells. In this study, the Human Protein Atlas (https://www.proteinatlas.org/) was utilized to determine the cell types expressing IGF2BP2 in the liver. The findings indicate that IGF2BP2 expression in the liver is primarily localized in vascular endothelial cells, cholangiocytes, and HSCs (Fig. S1A). In a comprehensive dataset analyzing gene expression profiles of HSCs (GSE67664), it was observed that the expression level of *IGF2BP2* was higher in activated HSCs compared to quiescent HSCs (Fig. S1B).

The LX-2 cell line, which is derived from human HSCs, has been extensively studied and characterized. It displays key features related to fibrosis [26]. Consequently, we investigated the expression of *IGF2BP2* in activated HSCs utilizing the LX-2 cell line. It has been shown that HSCs exhibit an active pro-fibrotic phenotype when grown in Dulbecco’s modified Eagle’s medium (DMEM) containing 10% fetal bovine serum (FBS) [27]. Next, we utilized a 10% FBS-stimulated model to detect *IGF2BP2* expression. We observed that the expression of *IGF2BP2* and *ACTA2* were increased in LX-2 cells following a 1 h incubation with 10% FBS, in comparison to cells cultured in control medium containing 2% FBS. (Fig. S1C).

In the model of LX-2 cell activation induced by 1 ng/mL transforming growth factor-β1 (TGF-β1), it was observed that the levels of *IGF2BP2* messenger RNA (mRNA) were markedly elevated following a 1 h exposure to TGF-β1, reaching their highest point after 6 h of treatment. (Fig. S1D). Moreover, the upregulation of *ACTA2* and *COL1α1* exhibited a temporal pattern and exhibited prolonged expression following TGF-β1 stimulation (Fig. S1D). Similarly, our study revealed that the protein expression levels of IGF2BP2 and α-SMA exhibited an increase following a 6 h induction of TGF-β1 (Fig. S1E). Together, these findings suggest that IGF2BP2 is upregulated in activated HSCs.

### Inhibition of IGF2BP2 blocks HSCs activation in vitro

**Fig. 3:**
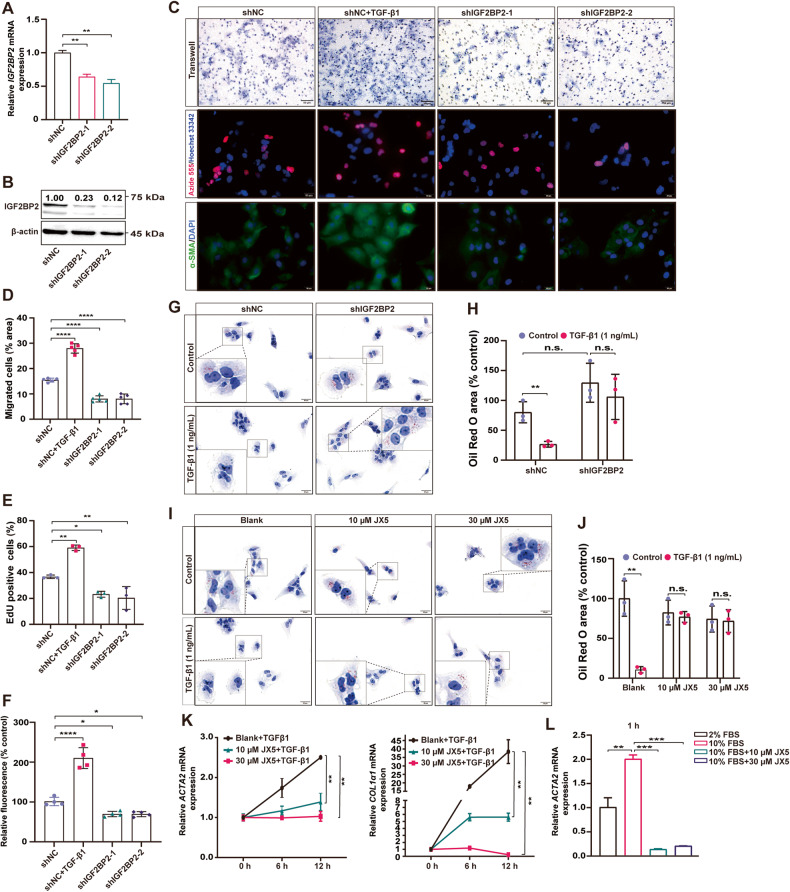
Inhibition of IGF2BP2 blocks HSCs activation in vitro. qRT-PCR (**A**) and immunoblotting (**B**) confirmed effective *IGF2BP2* KD by shRNA in LX-2 cells. Representative images (**C**) and bar plot (**D**–**F**) of cell migration assays (upper, scale bar, 50 µm), EdU cell proliferation assays (middle, scale bar, 20 µm), and immunofluorescence (bottom, scale bar, 20 µm) in *IGF2BP2* KD LX-2 cells, and shNC-transfected LX-2 cells treated with TGF-β1 as a positive control. Representative images (**G**) and bar plot (**H**) of oil red O staining in *IGF2BP2* KD LX-2 cells treated or untreated with TGF-β1 for 24 h (scale bar, 20 µm). Representative images (**I**) and bar graphs (**J**) of oil red O staining of LX-2 cells pretreated with JX5 (blank, 10 µM, 30 µM) for 8 h, followed by treatment or untreatment with TGF-β1 for 24 h (scale bar, 20 µm). **K** *ACTA2* and *COL1α1* mRNA expression in LX-2 cells pretreated with JX5 (blank, 10 µM, 30 µM) for 8 h, followed by treatment with TGF-β1 for different time points (0, 6, 12 h). **L** *ACTA2* mRNA expression in LX-2 cells cultured with 10% FBS treated with JX5 (blank, 10, 30 µM) for 1 h, and LX-2 cells cultured with 2% FBS as control. n.s., not significant; *P < 0.05, **P < 0.01, ***P < 0.001, ****P < 0.0001.

An altered phenotype of activated HSC has been demonstrated, including increased cell proliferation, migration to the site of injury, and expression of α-SMA [28, 29]. Given our findings that IGF2BP2 expression is upregulated in activated HSCs, we hypothesized that IGF2BP2 ablation hinders HSCs activation. To validate the hypothesis, we constructed cell lines with stable *IGF2BP2* knockdown (KD) using short hairpin RNA (shRNA) in LX-2 cells (Fig. 3A, B). The results of the cell migration assay demonstrated that the activation of LX-2 cells induced by TGF-β1 led to an increase in cell migration. Cell migration was blocked in *IGF2BP2* KD cells compared to cells transfected with sh-NC (Fig. 3C, D). By performing the 5-Ethynyl-2′-Deoxyuridine (EdU) incorporation assay, it was observed that around 60% of LX-2 cells stimulated by TGF-β1 exhibited labeling through EdU signaling, thereby suggesting a notable increase in the proliferation rate of LX-2 cells. Nevertheless, subsequent treatment of shIGF2BP2 resulted in a decrease in the proportion of EdU-positive cells (Fig. 3C, E). Furthermore, the inhibition of *IGF2BP2* resulted in the suppression of α-SMA expression, as demonstrated by immunofluorescence analysis (Fig. 3C, F).

In a state of quiescence, HSCs store retinol within their cytoplasm. However, upon activation, HSCs undergo morphological changes characterized by an enlarged cytosol and the depletion of lipid droplets [30]. Subsequently, oil red O staining was conducted to visualize the generation of lipid droplets in LX-2 cells. Our results indicate that TGF-β1 induction markedly attenuated lipid droplet production in LX-2 cells, and importantly, shIGF2BP2 treatment reversed this effect of TGF-β1 (Fig. 3G, H). In conclusion, the role of *IGF2BP2* in LX-2 cell proliferation, migration, α-SMA expression, and lipid droplet production was revealed. Together, these results suggest that inhibition of *IGF2BP2* blocks HSCs activation and permits fibroblasts to transform into a quiescent phenotype.

Recently, a novel small-molecule inhibitor of IGF2BP2, named JX5, has been characterized and shown to exert activity by binding to IGF2BP2 [31]. As expected, we observed that both 10 µM and 30 µM JX5 treatments reversed TGF-β1-induced lipid droplet reduction (Fig. 3I, J). Moreover, our study revealed that the treatment of JX5 effectively counteracted the progressive increase of *ACTA2* and *COL1α1* induced by TGF-β1, achieving complete reversal within a 12 h (Fig. 3K). Consistently, in the 10% FBS-stimulated HSCs activation model, JX5 treatment reversed the upregulation of *ACTA2* expression (Fig. 3L). These results reaffirm that inhibition of IGF2BP2 blocks HSCs activation.

### IGF2BP2 is involved in the ECM pathway and glycolytic metabolic processes

**Fig. 4:**
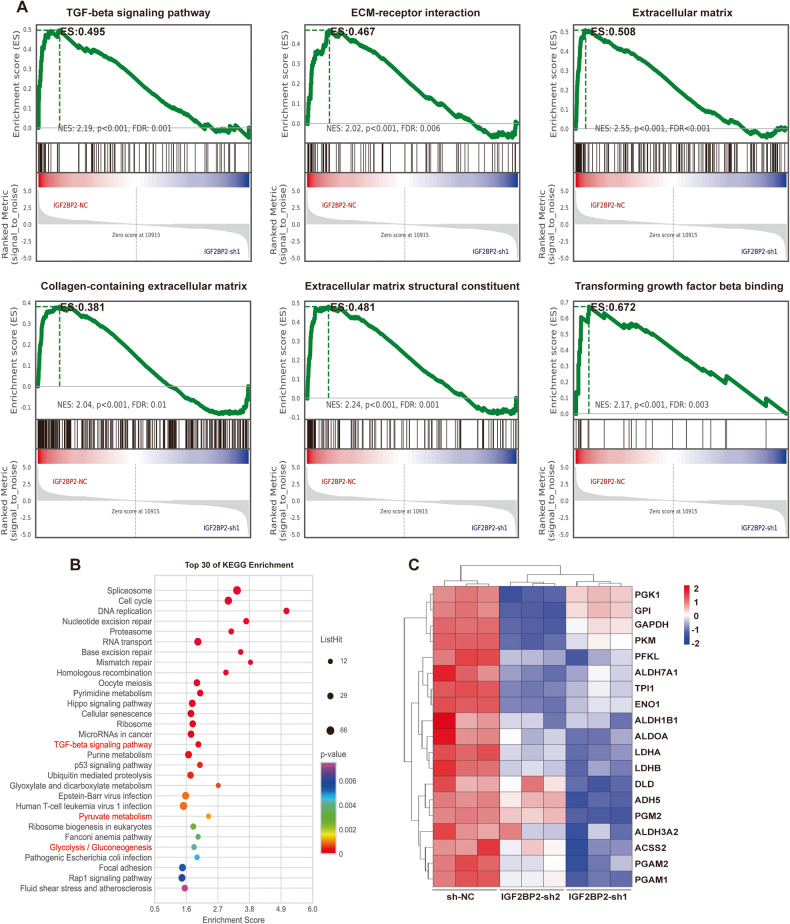
IGF2BP2 is involved in the ECM pathway and glycolytic metabolic processes. **A** Performing GSEA enrichment analysis in *IGF2BP2* KD LX-2 cells to demonstrate enrichment signaling pathways or biological processes involved in IGF2BP2. **B** Bubble diagram showing KEGG enrichment analysis in *IGF2BP2* KD LX-2 cells. The y-axis is for KEGG terms. The size and color of the bubbles represent the number and significance of genes associated with the term, respectively. **C** Heatmap representation of glycolysis/gluconeogenesis processes-related genes differentially expressed in *IGF2BP2* KD LX-2 cells. The color bar indicates the value of the Z-transformed expression.

To investigate the pathways and mechanisms through which IGF2BP2 facilitates the activation of HSCs, we conducted RNA sequencing (RNA-seq) analysis to compare the transcriptomes of LX-2 cells transfected with shNC and shIGF2BP2. Enrichment analysis was performed by running Gene Set Enrichment Analysis (GSEA), with Kyoto Encyclopedia of Genes and Genomes (KEGG) pathway gene sets serving as the classification criteria. This analysis identified several intersecting KEGG pathways, including the TGF-beta signaling pathway (normalized enrichment score (NES) = 2.19, *p* < 0.001) and ECM-receptor interaction (NES = 2.02, *p* < 0.001) in shNC and shIGF2BP2 transfected LX-2 cells (Fig. 4A). Moreover, our analysis revealed that Gene ontology (GO) was employed as a classification criterion to enhance the composition of the ECM (NES = 2.55, *p* < 0.001), collagen-containing ECM (NES = 2.04, *p* < 0.001), ECM structural constituent (NES = 2.24, *p* < 0.001), and TGF-beta binding (NES = 2.17, *p* < 0.001) (Fig. 4A). These findings once again provide evidence for the strong correlation between IGF2BP2 and liver fibrosis, highlighting its significant contribution to the development of liver fibrosis.

In our analysis of GO, IGF2BP2 was involved in the regulation of RNA binding, mRNA processing, and mRNA stability, which is consistent with the previously reported biological functions of IGF2BP2 [32] (Fig. S2). KEGG analysis of the differentially expressed genes (DEGs) showed that IGF2BP2 was mainly involved in the TGF-beta signaling pathway, pyruvate metabolism, and glycolysis/gluconeogenesis processes (Fig. 4B). Moreover, a notable differential expression of crucial glycolytic genes, namely *PGK1*, *PKM*, *PFKL*, *ENO1*, *ALDOA*, *LDHA*, and *LDHB*, was observed in LX-2 cells transfected with shNC and shIGF2BP2 (Fig. 4C). These data indicate that IGF2BP2 may play a role in the regulation of glycolysis-related genes and have an impact on liver fibrogenesis through its involvement in glycolytic metabolism.

Lactate, the final byproduct of glycolytic metabolism, was examined in the serum of mice afflicted with liver fibrosis induced by CCl_4_. The findings revealed a notable increase in lactate levels within the serum of mice suffering from fibrosis (Fig. S3A). Additional investigations revealed a reduction in lactate levels within cells that underwent *IGF2BP2* KD (Fig. S3B). Significantly, the depletion of IGF2BP2 resulted in an increase in glucose levels in the medium, indicating that the uptake of glucose is hindered in the absence of IGF2BP2 (Fig. S3C). Furthermore, we observed reduced Adenosine triphosphate (ATP) production in *IGF2BP2* KD cells (Fig. S3D). Consistently, the treatment of JX5 to cells resulted in a reduction in lactate levels and a decline in ATP production (Fig. S3E, F). The data presented in this study indicate a significant impact of IGF2BP2 on glycolytic metabolism and the regulation of lactate levels.

### IGF2BP2 regulates glycolytic metabolism by increasing *ALDOA* expression

**Fig. 5:**
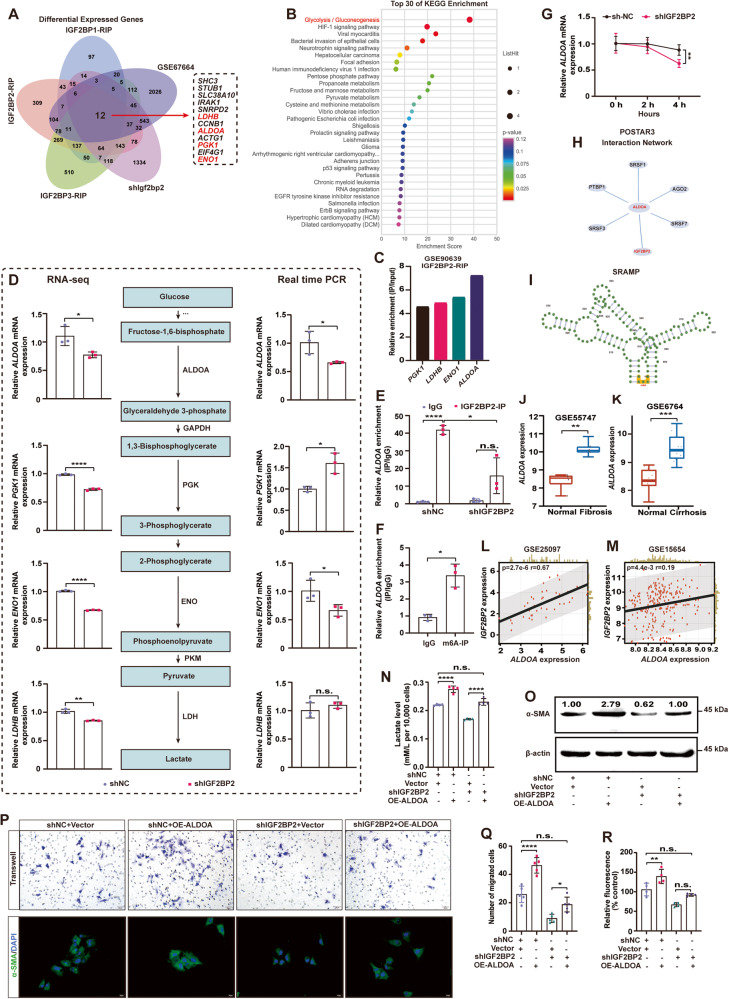
IGF2BP2 regulates glycolytic metabolism by increasing *ALDOA* expression. **A** Venn diagram showing screening strategies for candidate downstream targets of IGF2BP2. **B** Bubble diagram showing Kyoto Encyclopedia of Genes and Genomes (KEGG) enrichment analysis of candidate target genes screened by (**A**). The y-axis is for KEGG terms. The size and color of the bubbles represent the number and significance of genes associated with the term, respectively. **C** Expression of initially selected downstream targets (*PGK1*, *LDHB*, *ENO1*, and *ALDOA*) of IGF2BP2 in the GSE90639 dataset. **D** Expression of selected downstream targets (*PGK1*, *LDHB*, *ENO1*, and *ALDOA*) detected in RNA-seq and qRT-PCR, and metabolic schematic of selected targets in the glycolytic pathway. **E** RIP assay using IGF2BP2 antibody in *IGF2BP2* KD or control LX-2 cells. **F** RIP assay using m^6^A antibody in LX-2 cells. **G** *ALDOA* mRNA half-life in *IGF2BP2* KD or control LX-2 cells. **H** Interactions between IGF2BP2 and *ALDOA* were predicted based on POSTAR3 (http://postar.ncrnalab.org). **I** High-confidence m^6^A modification sites on *ALDOA* were predicted from SRAMP (www.cuilab.cn/). **J** *ALDOA* expression in CCl_4_-induced liver fibrosis and normal control from GSE55747. **K** *ALDOA* expression in liver cirrhosis and normal control from GSE6764. **L**, **M** Correlation analysis of *IGF2BP2* expression with *ALDOA* expression in cirrhotic samples from GSE25097 (*n* = 40) and GSE15654 (n = 216). **N-R** Measurement of lactate levels (**N**), α-SMA expression, and migration capacity (upper of Fig. 5P, scale bar, 50 µm) in LX-2 cells after co-transfection of *ALDOA* overexpression vector (OE-*ALDOA*) or control vector (Vector) in *IGF2BP2* KD (shIGF2BP2) and control (shNC) cells. α-SMA expression was measured using immunoblotting (**O**) and immunofluorescence (bottom of Fig. 5P, scale bar, 20 µm). There are bar graphs showing the migration capacity (**Q**) and the fluorescence intensity of α-SMA expression (**R**) in LX-2 cells. n.s. not significant; *P < 0.05, **P < 0.01, ***P < 0.001, ****P < 0.0001.

The IGF2BP2 protein has been reported to stabilize m^6^A-modified mRNAs [25]. We focused on positively regulated genes in screening strategies for downstream targets of IGF2BP2. Combining the dataset of RNA immunoprecipitation (RIP) performed by IGF2BP1/2/3 (GSE90639), the RNA-seq data we performed in *IGF2BP2* KD cells, and the highly expressed genes in activated HSCs (GSE67664), a total of 12 overlapping genes were obtained (Fig. 5A). Subsequently, a KEGG enrichment analysis was conducted on the 12 genes that exhibited overlap, revealing their predominant involvement in the metabolic processes of glycolysis and gluconeogenesis (Fig. 5B). This finding aligns with our prior data. Based on our strategy for screening downstream targets and the analysis of glycolytic pathways, we initially identified four genes (*ALDOA*, *PGK1*, *ENO1*, and *LDHB*) that are involved in glycolytic metabolism and show significant enrichment in the IGF2BP2 RIP according to the GSE90639 dataset (Fig. 5C), as potential targets of IGF2BP2. Given that IGF2BP2 has been shown to have a positive regulatory effect on the expression of target genes, we proceeded to investigate the impact of *IGF2BP2* KD on the expression of these four genes using qRT-PCR. Unexpectedly, our findings revealed that only two of the genes, namely *ALDOA* and *ENO1*, exhibited a decrease in expression following *IGF2BP2* KD (Fig. 5D).

To gain a deeper understanding of the downstream targets of IGF2BP2, we conducted a RIP assay. The results of this assay revealed a notable increase in the expression of *ALDOA* following incubation with the IGF2BP2 antibody, as compared to the IgG control group. Furthermore, the enrichment of *ALDOA* was found to be compromised upon knockdown of *IGF2BP2* (Fig. 5E). Likewise, the enrichment of *ALDOA* was achieved through incubation with the m^6^A antibody (Fig. 5F). Importantly, RNA stability assays revealed that the half-life of *ALDOA* RNA was shortened in cells with *IGF2BP2* KD after 4 h of actinomycin D treatment (Fig. 5G). According to the findings from POSTAR3 (http://postar.ncrnalab.org), it was predicted that there are significant interactions between IGF2BP2 and *ALDOA* (Fig. 5H). High-confidence m^6^A modification sites on *ALDOA* predicted from SRAMP (www.cuilab.cn/) (Fig. 5I). These findings collectively present compelling evidence supporting the direct binding and regulatory role of IGF2BP2 on *ALDOA*.

### Enhanced *ALDOA* expression rescues *IGF2BP2* KD-mediated inhibition of LX-2 cells activation

To further clarify the biological function of *ALDOA* in liver fibrosis, an analysis of the GSE55747 dataset revealed a notable upregulation of *ALDOA* expression in fibrotic liver tissues (Fig. 5J). Similarly, *ALDOA* expression was significantly increased in the livers of cirrhotic patients, according to the GSE6764 dataset (Fig. 5K). Further correlation pattern analysis in cirrhotic samples confirmed that *IGF2BP2* expression was positively linear with *ALDOA* in the GSE25097 (*p* = 2.7e-6, *r* = 0.67) and GSE15654 (*p* = 4.4e-3, *r* = 0.19) datasets (Fig. 5L, M).

To further determine the biological function of IGF2BP2 in driving HSCs activation through the regulation of *ALDOA* expression, we performed rescue experiments by enhancing *ALDOA* expression in *IGF2BP2* KD LX-2 cells. As expected, enhanced *ALDOA* expression effectively counteracted the inhibitory effects of *IGF2BP2* KD on LX-2 cell migration and the reduction of α-SMA expression., which are considered to be phenotypes of activated HSCs (Fig. 5O–R). In addition, enhanced *ALDOA* expression reversed the *IGF2BP2* KD-mediated decrease in lactate levels (Fig. 5N). Overall, our data reveal that *ALDOA* is a functionally critical target of IGF2BP2. The regulation of *ALDOA* expression by IGF2BP2 contributes to the overall biological function of IGF2BP2 in glycolytic metabolism and the activation of HSCs.

### Histone lactylation is essential in HSCs activation

**Fig. 6:**
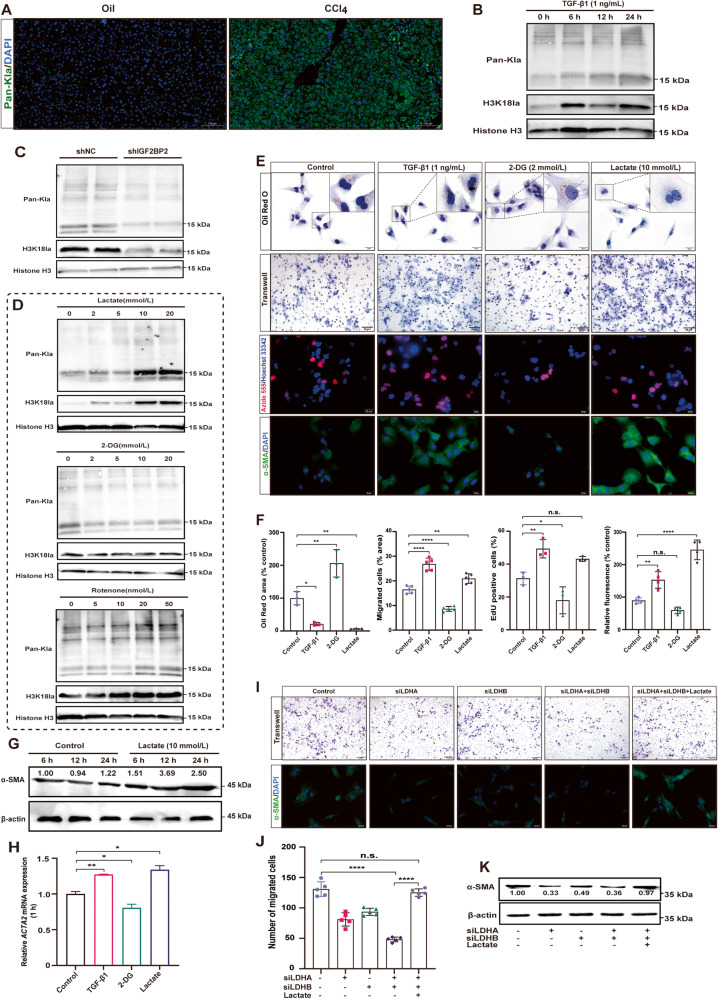
Histone lactylation is essential in HSCs activation. **A** Global lactylation levels in liver sections from CCl_4_-induced liver fibrosis mice and healthy mice were visualized by immunofluorescence (scale bar, 100 µm). **B** Immunoblotting showing global lactylation and H3K18la levels after TGF-β1-induced LX-2 cells for different times (0, 6, 12, 24 h). **C** Immunoblotting shows global lactylation and H3K18la levels in LX-2 cells with *IGF2BP2* KD. **D** Immunoblotting shows global lactylation and H3K18la levels in LX-2 cells treated with different concentrations of lactate, 2-DG, or rotenone for 24 h. Representative images (**E**) and bar graphs (**F**) showing lipid droplets, migration capacity, cell proliferation, and α-SMA expression after treatment of LX-2 cells with TGF-β1, 2-DG, or lactate, as detected by oil red O staining, cell migration assays, EdU cell proliferation assays, and immunofluorescence, respectively. **G** Immunoblotting showed expression of α-SMA at 10 mmol/L exogenous lactate supplementation for different times (6 h, 12 h, 24 h). **H** qRT-PCR verified the expression of *ACTA2* at 1 h of 2-DG, lactate treatment. Representative images (upper of **I**, scale bar, 50 µm) and bar plot (**J**) showing cell migration in LX-2 cells silenced for *LDHA* and *LDHB*. Representative images showing α-SMA expression (bottom of Fig. 6I, scale bar, 20 µm) in LX-2 cells silenced for *LDHA* and *LDHB*. **K** Immunoblotting shows α-SMA expression in LX-2 cells silenced for *LDHA* and *LDHB*. n.s. not significant; *P < 0.05, **P < 0.01, ****P < 0.0001.

Our data indicate that activated HSCs exhibit active glycolysis and lead to the accumulation of lactate, which is a substrate for histone lactylation. Next, we investigated the level of histone lactylation in liver fibrosis and its role in the pathogenesis of liver fibrosis. Immunofluorescence revealed a notable increase in the global lactylation level within liver fibrosis tissues induced by CCl_4_ in mice, in comparison to healthy liver tissues (Fig. 6A). There was an observed elevation in the global lactylation and H3K18la levels following a 24 h treatment of LX-2 cells with 1 ng/mL TGF-β1, especially the increase in H3K18la, which was more significant (Fig. 6B). Importantly, IGF2BP2 further affects histone lactylation by regulating lactate production, and our results observed both global lactylation and H3K18la levels were reduced in cells with *IGF2BP2* KD (Fig. 6C). These data support the idea that activated HSCs are characterized by elevated levels of lactylation, which may be involved in the activation of HSCs and the pathogenesis of liver fibrosis.

To assess whether histone lactylation functions in LX-2 activation, we used several compounds to alter the global intracellular lactylation levels. Obviously, 10 mmol/L exogenous lactate supplementation significantly enhanced global lactylation and H3K18la levels (Fig. 6D). 2-deoxy-D-glucose (2-DG), a recognized inhibitor of glycolysis, was observed to inhibit global lactylation levels (Fig. 6D). Rotenone, an inhibitor of mitochondrial respiratory chain complex I, increases global lactylation and H3K18la levels in cells (Fig. 6D). Here, we performed oil red O staining, cell migration assays, and EdU cell proliferation assays to assess the effect of altered lactylation levels on LX-2 cell activation. Next, we observed that inhibition of histone lactylation using 2-DG effectively enhanced the production of intracellular lipid droplets in LX-2 cells. Additionally, it impeded the migration and proliferation of LX-2 cells (Fig. 6E, F).

In order to further investigate the correlation between histone lactylation and the activation of HSCs, exogenous lactate supplementation was employed. The findings of our study indicate a noteworthy decrease in lipid droplets and an elevation in cell migration in LX-2 cells following the augmentation of histone lactylation through the treatment of lactate. However, no substantial variance in LX-2 cell proliferation was observed when lactate was utilized, potentially attributable to the robust proliferation of LX-2 cells cultured in vitro (Fig. 6E, F). Furthermore, the application of lactate was found to augment the expression of α-SMA, as demonstrated by immunofluorescence analysis (Fig. 6E, F). Furthermore, lactate supplementation activated α-SMA expression, as further confirmed by immunoblotting and qRT-PCR (Fig. 6G, H). Conversely, the application of 2-DG led to a reduction in α-SMA expression (Fig. 6H).

Inhibition of lactate dehydrogenase (*LDH*) has been reported to be sufficient to reduce global lactylation levels [10, 11, 33]. In order to further validate the association between lactylation and the activation of HSCs, we proceeded to silence *LDHA* and *LDHB*. As expected, our results indicate that the combined silencing of *LDHA* and *LDHB* triggered a significant inhibition of LX-2 cell migration and α-SMA expression (Fig. 6I–K). Interestingly, supplementation of exogenous lactate to cells co-silenced by *LDHA* and *LDHB* restored cell migration and α-SMA expression (Fig. 6I–K). Taken together, these results emphasize the essential role of histone lactylation in HSCs activation.

### Inhibition of histone lactylation ameliorates liver fibrosis in vivo

**Fig. 7:**
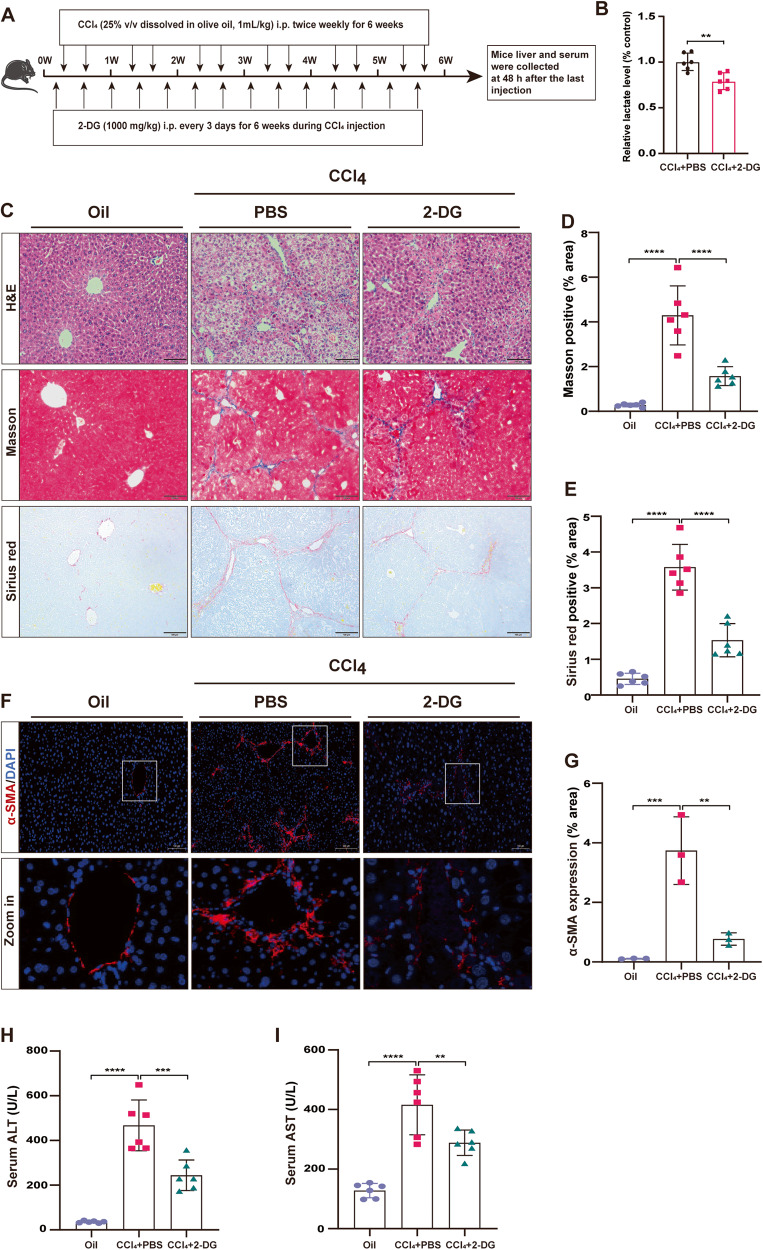
Inhibition of histone lactylation ameliorates liver fibrosis in vivo. **A** Schematic illustration of the experimental design of 2-DG (1000 mg/kg) for the treatment of CCl_4_-induced liver fibrosis in mice. **B** Lactate levels in the serum of mice treated according to (**A**) (*n* = 6). Representative images (**C**) and bar plot (**D**, **E**) of H&E staining (upper), Masson’s trichrome staining (middle), and sirius red staining (bottom) in mouse liver sections treated according to (**A**) (n = 6, scale bar, 100 µm). Representative images (**F**) and bar plot (**G**) of α-SMA expression (upper, *n* = 3, scale bar, 100 µm) visualized by immunofluorescence in mouse liver sections treated according to (**A**). Serum levels of ALT (**H**) and AST (**I**) in mice treated according to (**A**). **P < 0.01, ***P < 0.001, ****P < 0.0001.

Considering that the use of 2-DG inhibited global lactylation levels and HSCs activation in vitro, next, we aimed to assess the alleviation of liver fibrosis by intraperitoneal injection of 2-DG (Fig. 7A). Significantly, 2-DG attenuated serum lactate levels in mice with CCl_4_-induced liver fibrosis (Fig. 7B). In addition, our results showed reduced areas of collagen deposition and attenuated liver injury in the livers of 2-DG-treated mice, as evidenced by H&E staining, Masson’s trichrome staining, and sirius red staining (Fig. 7C–E). Our immunofluorescence results showed that treatment with 2-DG effectively inhibited α-SMA expression in the mouse liver (Fig. 7F, G). Furthermore, analysis of ALT and AST levels indicated that treatment with 2-DG resulted in the restoration of liver function in liver fibrosis mice (Fig. 7H, I). In conclusion, these data collectively suggest that the blockade of lactylation alleviates liver fibrosis.

## Discussion

Liver fibrosis is reversible after the removal of the causative agent. Although promising progress has been made in the study of the mechanisms of liver fibrosis onset and reversal over the past decades, the understanding of some of the mechanisms is not yet fully clear. Currently, the role of m^6^A in the pathogenesis of liver fibrosis is being revealed [34]. This work shows that IGF2BP2, a newly identified m^6^A reader, is upregulated in liver fibrosis and activated HSCs. We show here that the use of RNA infection and small-molecule compound inhibition of IGF2BP2 could block HSCs activation in vitro. Notably, mice lacking IGF2BP2 were protected from liver fibrosis in vivo. These data further extend the evidence that the involvement of m^6^A is essential for liver fibrosis and that m^6^A provides new insights into the understanding of the molecular mechanisms of liver fibrosis and may represent a new therapeutic approach.

Although there may be some differences in the pathology and molecular mechanisms of liver fibrosis caused by multiple etiologies, we need to be cautious when using CCl_4_-induced liver fibrosis models to extrapolate or explain the true state of liver fibrosis in humans. However, recent studies have shown that IGF2BP2^−/−^ mice exhibit resistance to diet-induced obesity and fatty liver [19]. In addition, it has been shown that inhibition of IGF2BP2 protects against diet-induced hepatic steatosis [20]. Another study showed that IGF2BP2 knockdown effectively inhibited CCl_4_-induced liver fibrosis, which is consistent with our data [21]. Although it represents potential, however, the suitability of IGF2BP2 as a therapeutic target for different etiologies of liver fibrosis requires further validation.

Innovatively, this work reveals a novel regulatory pathway for IGF2BP2 in HSCs. Our results suggest that IGF2BP2 enhances the expression of *ALDOA*, a key target in the glycolytic pathway. We observed that inhibition of IGF2BP2 impaired lactate production in LX-2 cells, and enhanced *ALDOA* expression rescued the IGF2BP2-mediated reduction in lactate production. Importantly, enhanced Aldoa expression rescued the *IGF2BP2* KD-mediated inhibition of LX-2 cell activation. It has been reported that glycolysis predisposes HSCs to activation, as evidenced by cell migration and extracellular matrix deposition [7]. From a biogenesis point of view, glycolysis provides a continuous energy supply for the activation of HSCs to accommodate the energy requirements for HSCs proliferation and differentiation [35]. Accumulating evidence suggests that interfering with glycolysis in HSCs can effectively restore their quiescent phenotype and has been shown to be a promising therapeutic target for anti-liver fibrosis [8, 36–38]. An outstanding issue, however, is that dysregulation of multiple glycolytic genes, including pyruvate kinase M2 (*PKM2*) [39], hexokinase 2 (*HK2*) [33], and fructose-2,6-bisphosphatase-3 (*PFKFB3*) [7], has been identified in liver fibrosis, which complicates understanding the relationship between liver fibrosis and glycolysis. It is unclear whether IGF2BP2-mediated glycolytic metabolism plays a central role in the process of liver fibrosis or is the result of joint involvement with other glycolytic genes.

Activated HSCs exhibit active glycolysis, producing large amounts of lactate as a substrate for histone lactylation. The role of histone lactylation in the mechanism of liver fibrosis remains to be elucidated. Here, we found increased levels of global lactylation in the livers of mice with CCl_4_-induced liver fibrosis, and increased levels of global lactylation and H3K18la were observed in TGF-β1-treated LX-2 cells. Importantly, correction of aberrant lactylation or exogenous supplementation of lactate was observed to alter the activation phenotype of LX-2 cells, as expected. A recent study showed an enrichment of H3K18la in activated HSCs. Interestingly, interfering with lactate production reduced H3K18la levels and inactivated HSCs [33]. These results are consistent with our data, although different cell types were used. Lactylation sites on different lysine residues in core histones have also been identified. A recent study has shown that H4K12la is enriched at the promoters of glycolytic genes and enhances glycolytic activity by promoting the transcription of glycolytic genes. This positive feedback regulation exacerbates microglia dysfunction in Alzheimer’s disease [40]. In addition, multiple lactylation sites have been identified on non-histone proteins as well, suggesting that lactylation is involved in a wider range of biological functions than just transcriptional regulation [41, 42]. The role of these lactylations identified at different sites of core histones as well as non-histone lactylation in the activation of HSCs and liver fibrosis deserves further investigation.

The present study has several limitations. First, despite sufficient in vitro investigations, it is imperative to employ an in vivo methodology that specifically focuses on haematopoietic stem cells, an aspect that was absent in our research. Furthermore, the AAV8 vector demonstrates a tropism for hepatocytes, and our investigators have noted favorable outcomes with its utilization. Nevertheless, the mechanism by which IGF2BP2 operates via hepatocytes necessitates further elucidation. Another limitation is that, despite validating the direct interaction of IGF2BP2 with *ALDOA*, further testing is needed to see if IGF2BP2 binds to the m^6^A site on *ALDOA* via KH1-KH4 domains, which would further deepen our understanding of the regulatory mechanism of IGF2BP2. In addition, lactate produced by activated HSCs may affect liver fibrosis through multiple links, and it would be interesting to explore the regulation of the hepatic inflammatory response by lactate as well as lactate-mediated crosstalk of HSCs with other cell types, such as hepatocytes, kupffer cells, and endothelial cells.

**Fig. 8:**
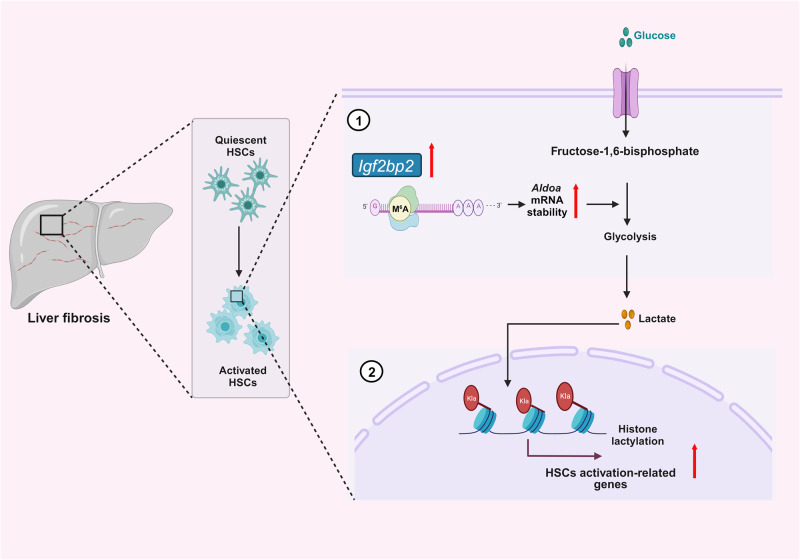
Schematic model of the mechanism of IGF2BP2 and histone lactylation in liver fibrosis. IGF2BP2 is highly activated in fibrotic livers of mice. Mechanistically, IGF2BP2 enhances the stability of the m^6^A-modified transcript *ALDOA* in the glycolytic metabolic pathway, which is crucial for HSCs activation. In addition, lactate produced by activated HSCs via the glycolytic pathway serves as a substrate for lactylation, which plays an essential role in the activation of HSCs. Created with BioRender.com.

In conclusion, our study reveals the essential role of IGF2BP2 as an m^6^A-binding protein in the pathogenesis of liver fibrosis through the regulation of glycolytic metabolism and extends the potential role of lactylation in the activation of HSCs and liver fibrosis (Fig. 8). Importantly, our study highlights that targeting IGF2BP2 could be an effective strategy for anti-liver fibrosis therapy.

## Materials and methods

### Human samples

Human samples and data were acquired from the First Hospital of Lanzhou University. Liver samples were procured from unaffected tissue adjacent to surgically removed hepatic hemangiomas, as well as from sclerotic tissue adjacent to hepatocellular carcinoma (HCC). Serum samples were obtained from both healthy individuals and patients diagnosed with cirrhosis. All participants provided informed consent to partake in this investigation. The research protocol was granted ethical approval by the Ethics Committee of the First Hospital of Lanzhou University (LDYYLL2023-333).

### Animal experiments

Male C57BL/6 mice, aged 7 weeks and weighing between 18–22 g, were obtained from Gempharmatech Co., Ltd. in Chengdu, China. The mice were housed in a specific pathogen-free facility with controlled ventilation and a 12-h light/dark cycle. All animal procedures were conducted in compliance with the Regulations on the Management of Laboratory Animals and were approved by the Ethics Committee of the First Hospital of Lanzhou University (LDYYLL2023-333).

The liver fibrosis model induced by CCl_4_ was implemented according to the previously described method [43]. In summary, mice were administered sterile CCl_4_ (25% v/v dissolved in olive oil, 1 mL/kg) via intraperitoneal injection twice a week for a duration of 6 weeks. After a 48-h period following the final injection, the mice were euthanized in order to collect serum and liver tissue for subsequent analysis.

In order to investigate the impact of *IGF2BP2* deficiency on the mitigation of liver fibrosis, a series of treatments were administered to mice. Following a one-week period of acclimatization to their new surroundings, the mice were assigned to different groups of 6 mice each using blinding and randomization methods. These groups included a control group treated with non-specific shRNA known as AAV-NC, and a group treated with shRNA specifically targeting murine *IGF2BP2*, referred to as AAV-shRNA. The shRNA used in the study was developed by OBiO Technology (Shanghai, China). The sequences of mouse AAV-shRNA and AAV-NC were cloned into the pAAV-TBG-mcherry-3xFLAG vector for further packaging. AAV serotype 8 was further selected to construct AAV8. Purified AAV8 was diluted in sterile phosphate buffer saline (PBS) and delivered to the mice by tail vein injection. Following the AAV8 injection, the mice were subjected to CCl_4_ treatment after a period of 2 weeks. The specific AAV sequences and vector genomes (vg) that were introduced into mouse can be found in Table S1.

To verify the effect of inhibiting histone lactylation on alleviating liver fibrosis, a study was conducted on mice. Mice were injected with olive oil or CCl_4_ twice a week for 6 weeks. During the injection of CCl_4_, 2-DG (1000 mg/kg, TagerMol) was injected intraperitoneally every 3 days. Mice were sacrificed 48 h after the last injection.

### Histological analysis and serum biochemistry

Liver tissues that had been fixed with 4% paraformaldehyde and embedded in paraffin were subjected to deparaffinization in order to carry out staining procedures such as H&E staining, Masson’s trichrome staining, and sirius red staining. ImageJ was performed to analyze the stained area of liver tissue sections after Masson’s trichrome staining and sirius red staining. The levels of ALT and AST in the serum were determined using the instructions provided by the manufacturer (Nanjing Jiancheng Bioengineering Institute, Nanjing, China).

### Cell culture

The LX-2 cells were cryopreserved in the laboratory and authenticated using short tandem repeat analysis. The cells were cultured in DMEM (Gibco, USA) supplemented with 2% (v/v) FBS (HyClone, USA). The cells were maintained in a controlled environment at 37 °C with 5% CO_2_. Additionally, the cells were regularly screened for mycoplasma contamination.

### Cell transfection

To knockdown *IGF2BP2* in LX-2 cells, either a non-specific shRNA (referred to as shNC) or a gene-specific shRNA was introduced into the cells using the recommended protocol provided by the manufacturer (Genechem, Shanghai, China). This transfection was performed when the cells reached a confluency of 40–50%. Following transfection, stable cell lines were established by subjecting the cells to puromycin screening at a concentration of 2 µg/mL.

In order to disrupt the expression of *LDHA* and *LDHB* genes in LX-2 cells, small interfering RNAs (siRNAs) were designed and synthesized by GenePharma (Shanghai, China). Gene-specific siRNA and a scrambled negative control siRNA were introduced into LX-2 cells through transfection using GP-transfect-Mate, a transfection reagent provided by GenePharma, following the guidelines provided by the manufacturer. After a transfection period of 12 h, fresh growth media was supplemented, and the cells were harvested 48 h later.

To enhance the expression of *ALDOA*, LX-2 cells were subjected to transfection with *ALDOA* overexpression and negative control vectors using E-trans as per the instructions provided by the manufacturer (Genechem, Shanghai). The cells were cultured until they reached a confluency of 70–80%. The E-trans-DNA mixture (consisting of 1 μg vector, 3 µL E-trans, and 100 µL serum-free DMEM) was prepared for each well, followed by a 10 min incubation at room temperature. Subsequently, the mixture was transferred to the corresponding 12-well plates. After an incubation period of 48 h, the cells were collected for further analysis.

The sequences of all RNA interference products are presented in Table S2.

### ELISA

Serum IGF2BP2 levels were quantified in patients with cirrhosis (*n* = 46) and healthy individuals (*n* = 24) using ELISA kits obtained from CUSABIO (Wuhan, China). The ROC curves were generated utilizing the OmicStudio tools (https://www.omicstudio.cn/tool/58).

### Lactate level assays

The lactate concentration in the medium was assessed following the protocol provided by the l-lactic acid (LA) Colorimetric Assay Kit (Elabscience, Wuhan, China). LX-2 cells were cultured for 48 h, and the supernatants were collected and diluted with PBS. A 5 µL aliquot of the diluted sample was taken for each well, followed by the addition of 100 µL of enzyme working solution and 20 µL of chromogenic agent. The mixture was then incubated at 37 °C for 10 min. A stop solution was added to each well, and the absorbance was measured at 530 nm using a full-wavelength microplate reader. To account for variations in cell number, the lactate content was normalized using a cell counter (Nexcelom Bioscience, USA). A similar procedure was employed to determine lactate levels in serum.

### ATP assay and glucose assay

The ATP levels were measured using the ATP assay kit (Beyotime, China; Cat # S0026). The cells were lysed and the supernatant was collected after centrifugation at 12,000 g for 5 min. The ATP assay working solution (100 µL) was added to the assay wells and incubated at RT for 3 min to deplete background ATP. The supernatant (20 µL) was then added and chemiluminescence was detected using a microplate reader (BioTek, USA). The protein concentrations of the samples were determined using the bicinchoninic acid (BCA) method. The glucose assay was performed following the instructions of the kit (Beyotime, China; Cat # S0201S), and the glucose level was calculated using the standard curve.

### Cell migration assays

The cell migration assay was conducted following the established protocol [44]. A total of five random fields of view were chosen for the purpose of capturing photographs and migrated cell analysis. The area or number of migrated cells was analyzed using ImageJ.

### Oil red O staining

Oil red O staining was conducted using a modified oil red O staining Kit (Solarbio, China; Cat # G1263). To begin, cells were fixed with 4% paraformaldehyde and then covered with oil red O buffer for a duration of 5 min. Subsequently, the cells were stained at RT for 15 min by adding the oil red O staining solution. Following this, the cells were treated with oil red O differentiation solution and stained with drops of mayer hematoxylin solution. After thorough washing, the stained fat droplets within the cells were observed and photographed using a light microscope. Positively stained areas were analyzed using ImageJ and normalized by controls.

### EdU cell proliferation assays

To evaluate cell proliferation, the BeyoClick™ EdU Cell Proliferation Kit (Beyotime, China; Cat # C0075S) was employed. The cells were subjected to incubation at 37 °C for 30 min with a pre-warmed EdU working solution (10 M). Following EdU labeling, the cells were fixed with 4% paraformaldehyde for 15 min. Subsequently, the cells were washed three times with PBS containing 3% BSA and permeabilized with enhanced permeabilization buffer (Beyotime, Cat # P0097) for 15 min, followed by another round of three washes. Each well was then incubated in the dark at RT for 30 min with the click reaction solution, washed, and finally stained with Hoechst 33342 for nuclear visualization. Fluorescence microscopy was employed to capture images.

### Immunofluorescence

As described previously, immunofluorescence was performed on cells [45]. In brief, the cells were fixed and blocked, followed by incubation with an anti-α-SMA antibody (1:200, Signalway Antibody, USA; Cat # 40482-1) and a FITC-conjugated fluorescent secondary antibody (1:200, Proteintech, China; Cat # SA00003-1). The stained cells were then mounted with a 4′,6-diamidino-2-phenylindole (DAPI)-containing antifluorescent attenuation blocker and observed under a fluorescence microscope (Olympus IX73, Japan). Multiple random fields of view were selected and analyzed for quantification of fluorescence using ImageJ. For tissue immunofluorescence, the sections were deparaffinized and rehydrated, and the remaining steps were identical to those for cell immunofluorescence.

### RNA isolation and qRT-PCR

RNA extraction and qRT-PCR procedures were conducted following the methods described in a previous study [46]. β-actin was utilized as an internal control for normalization. The specific primer sequences used in the qRT-PCR analysis can be found in Table S3.

### RNA-seq and data analysis

Three samples each of LX-2 cells with or without *IGF2BP2* KD were collected, and total RNA was extracted using RNAiso Plus (Takara). The library was constructed, and PCR amplification was used for library fragment enrichment. The library was then subjected to quality control using the Agilent 2100 Bioanalyzer. Sequencing of the library was performed using the HiSeq 2500 System (Illumina, USA), with sequencing services provided by oebiotech (Shanghai, China). DEGs between groups were analyzed using the DEG-seq package of R software. GO and KEGG enrichment analysis of DEGs was performed using the GO database and the KEGG database and visualized by the oebiotech tool (https://cloud.oebiotech.cn/task/).

### RIP assay

The RIP assay was performed utilizing the RIP kit (BersinBio, Guangzhou, China). In brief, a total of 2 × 10^7^ LX-2 cells were collected and lysed. Following the removal of DNA, the lysate was incubated with RIP buffer containing anti-IGF2BP2 antibody (3 µg, Proteintech; Cat # 11601-1-AP) and normal rabbit control IgG (3 µg, Bioss Antibodies; Cat # bs-0295PC) at 4 °C for 16 h. Subsequently, RNA-protein complexes were incubated with protein A/G magnetic beads that had been equilibrated. After the elution of RNA, the immunoprecipitated RNA was extracted, and the qRT-PCR program was executed.

### RNA stability assays

LX-2 cells were subjected to treatment with actinomycin D (HY-17559, MedChemExpress) at a concentration of 5 µg/mL. Subsequently, an equal number of cells (1 × 10^6^) were harvested at specified time intervals (0, 2, and 4 h) using a cell counter. Following this, total RNA was extracted and subjected to qRT-PCR analysis after reverse transcription.

### Immunoblotting

Immunoblotting was conducted following the previously established protocol [47]. An internal control was employed using either an anti-β-actin antibody, anti-Histone-H3 antibody or an anti-GAPDH antibody. The primary antibodies utilized for immunoblotting are listed in Table S4.

### Statistical analyses

In the present investigation, the statistical analysis of the data was conducted using SPSS 22.0 (SPSS Inc.) and GraphPad Prism 9 (GraphPad, USA) software. Pearson correlation analysis and Spearman correlation analysis were employed to ascertain the correlation between the expression of the metrics included in the cirrhosis datasets and mice with liver fibrosis, respectively. Group comparisons of continuous variables, which were presented as mean ± standard deviation, were performed using either a two-tailed student’s *t*-test or one-way ANOVA. *p* < 0.05 was considered indicative of a statistically significant.

### Supplementary information

Checklist

IGF2BP2 is upregulated in activated HSCs.

GO enrichment analyses in IGF2BP2 KD LX-2 cells.

Inhibition of IGF2BP2 blocks lactate production in vitro

Supplementary Figure Legends

Mouse AAV sequences and vector genomes (vg)

Sequence information for all RNA interference in this study

The specific primer sequences used in the qRT-PCR analysis in this study

Primary antibodies used for immunoblotting

Original Data File

## Data Availability

The datasets presented in this study are available in online repositories. Raw RNA sequencing data available upon reasonable request.
