# Supplementary figures and images for: The m6A reader IGF2BP2 regulates glycolytic metabolism and mediates histone lactylation to enhance hepatic stellate cell activation and liver fibrosis
Source: Cell Death Dis. 2024 Mar 5;15(3):189. doi: 10.1038/s41419-024-06509-9 (PMC10914723; doi:10.1038/s41419-024-06509-9)

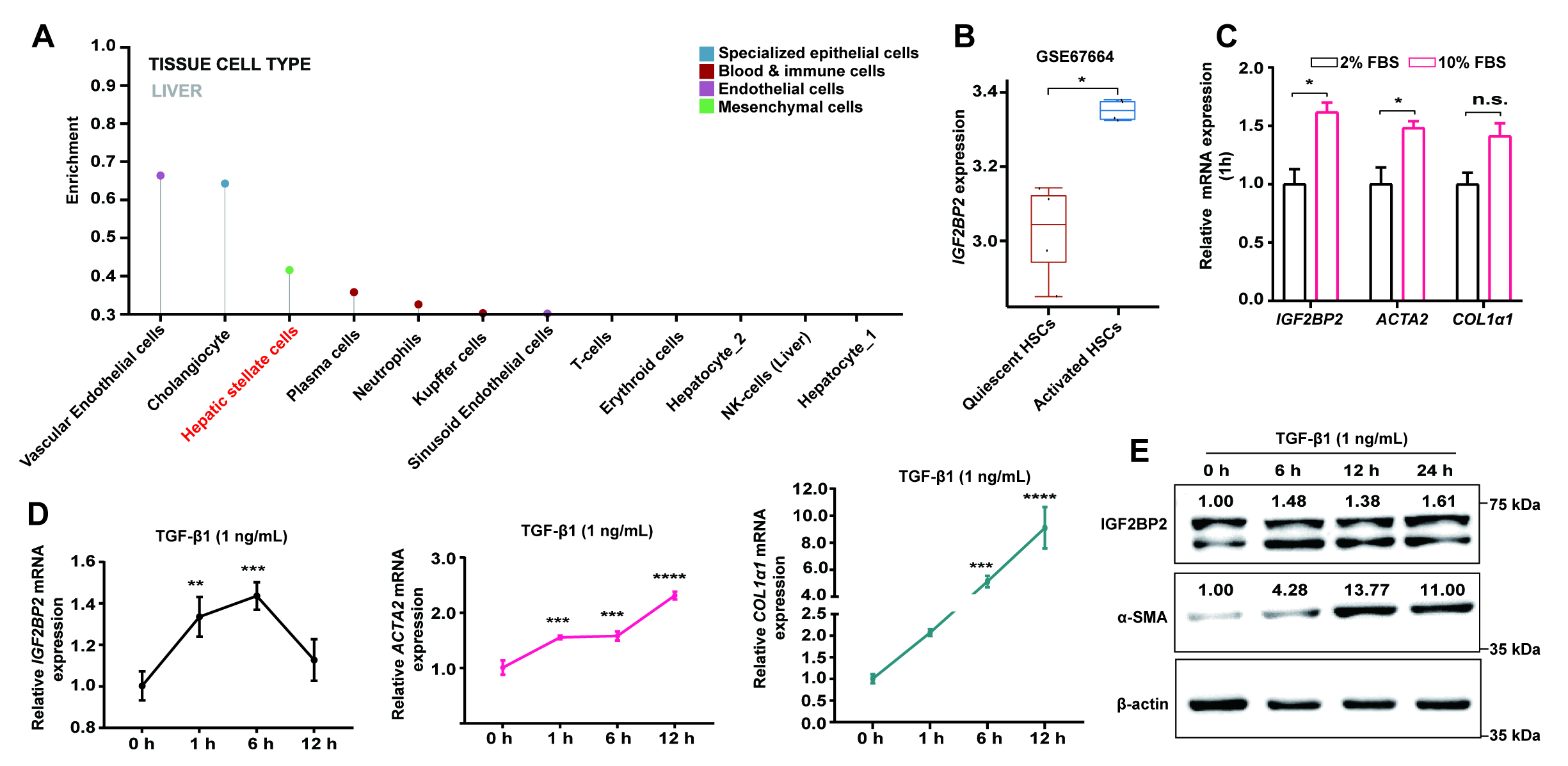

Supplement: Supplementary file 2 — IGF2BP2 is upregulated in activated HSCs. [file 41419_2024_6509_MOESM2_ESM.tif]

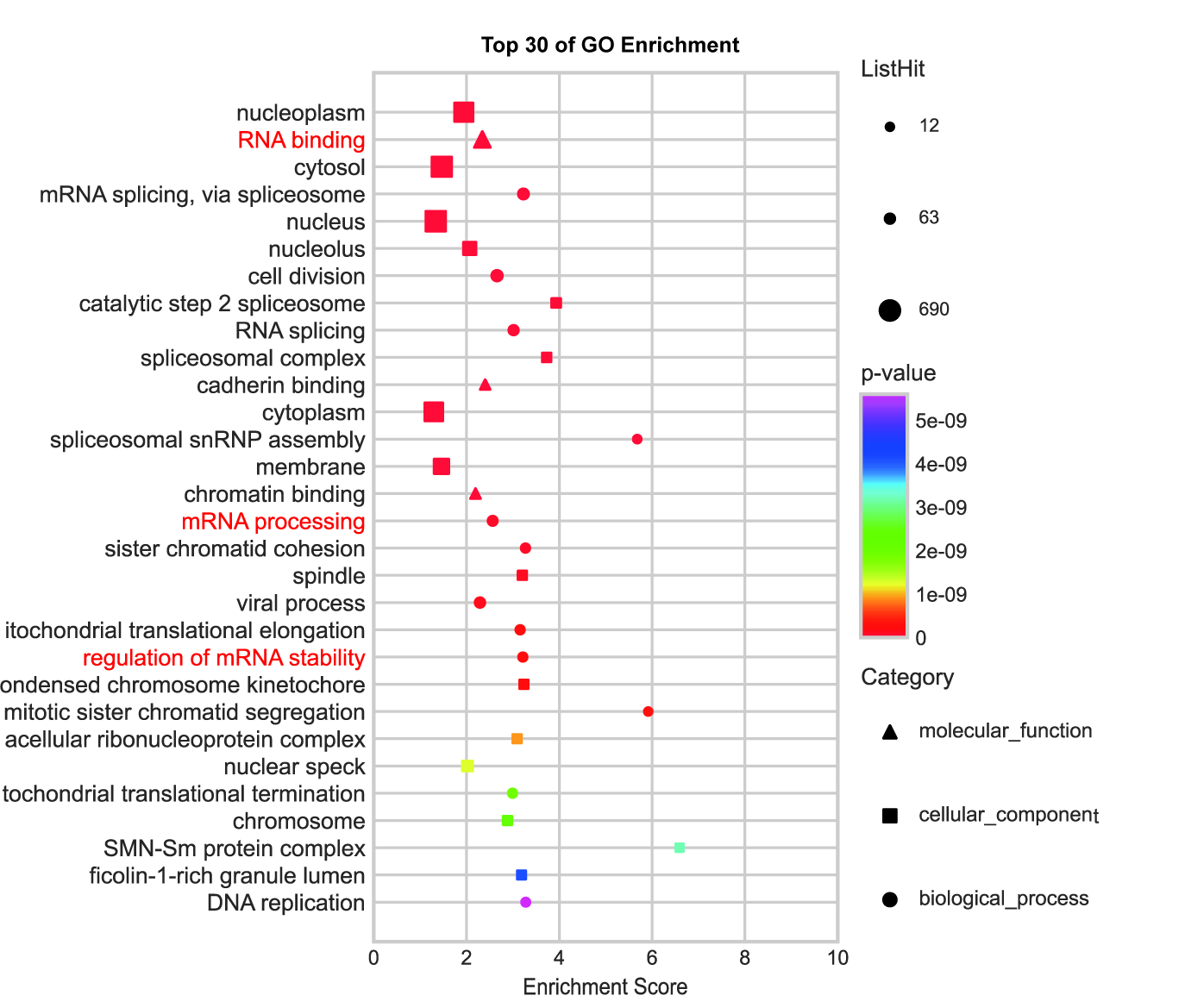

Supplement: Supplementary file 3 — GO enrichment analyses in IGF2BP2 KD LX-2 cells. [file 41419_2024_6509_MOESM3_ESM.tif]

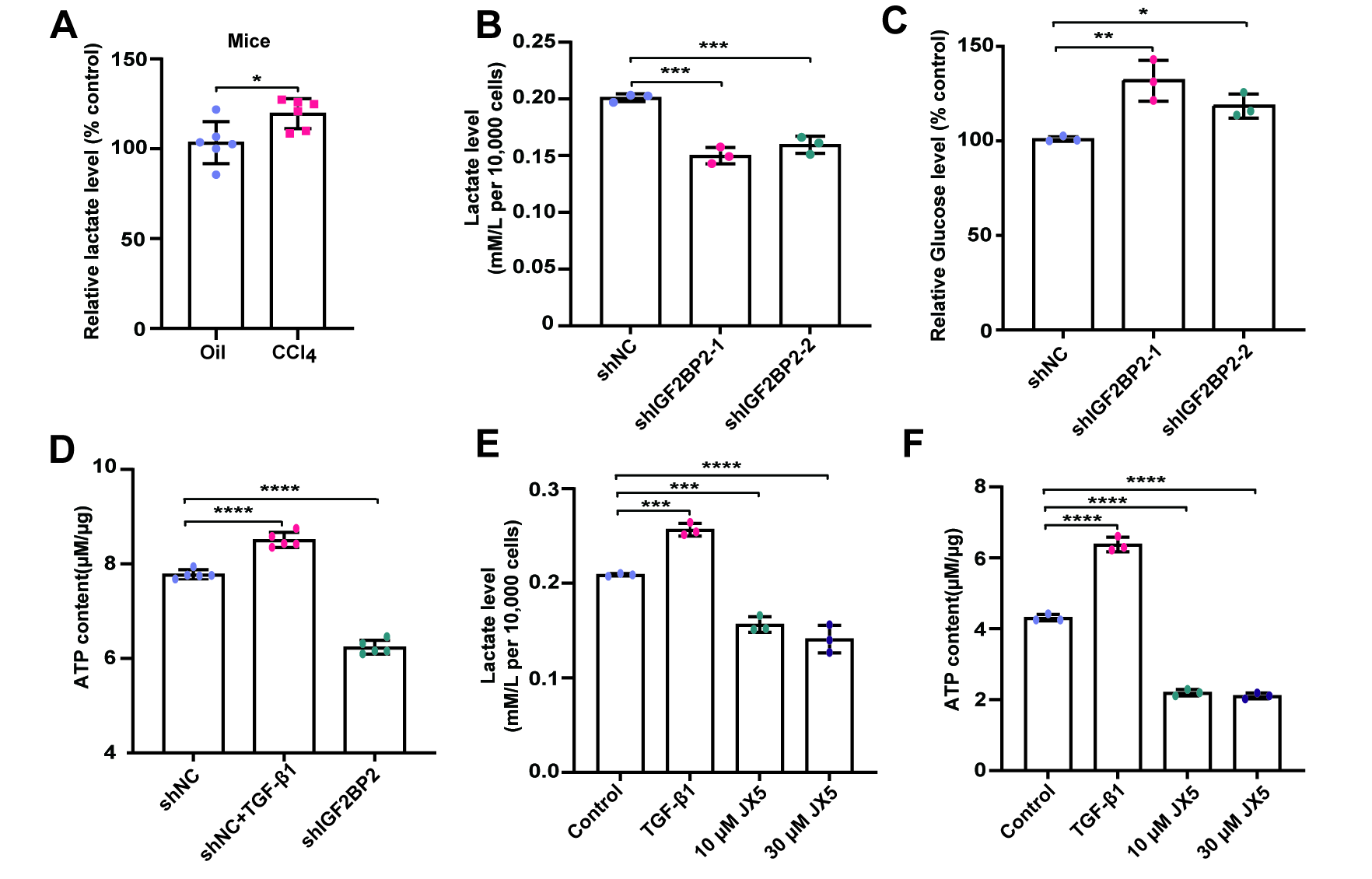

Supplement: Supplementary file 4 — Inhibition of IGF2BP2 blocks lactate production in vitro [file 41419_2024_6509_MOESM4_ESM.tif]

Figure 1J

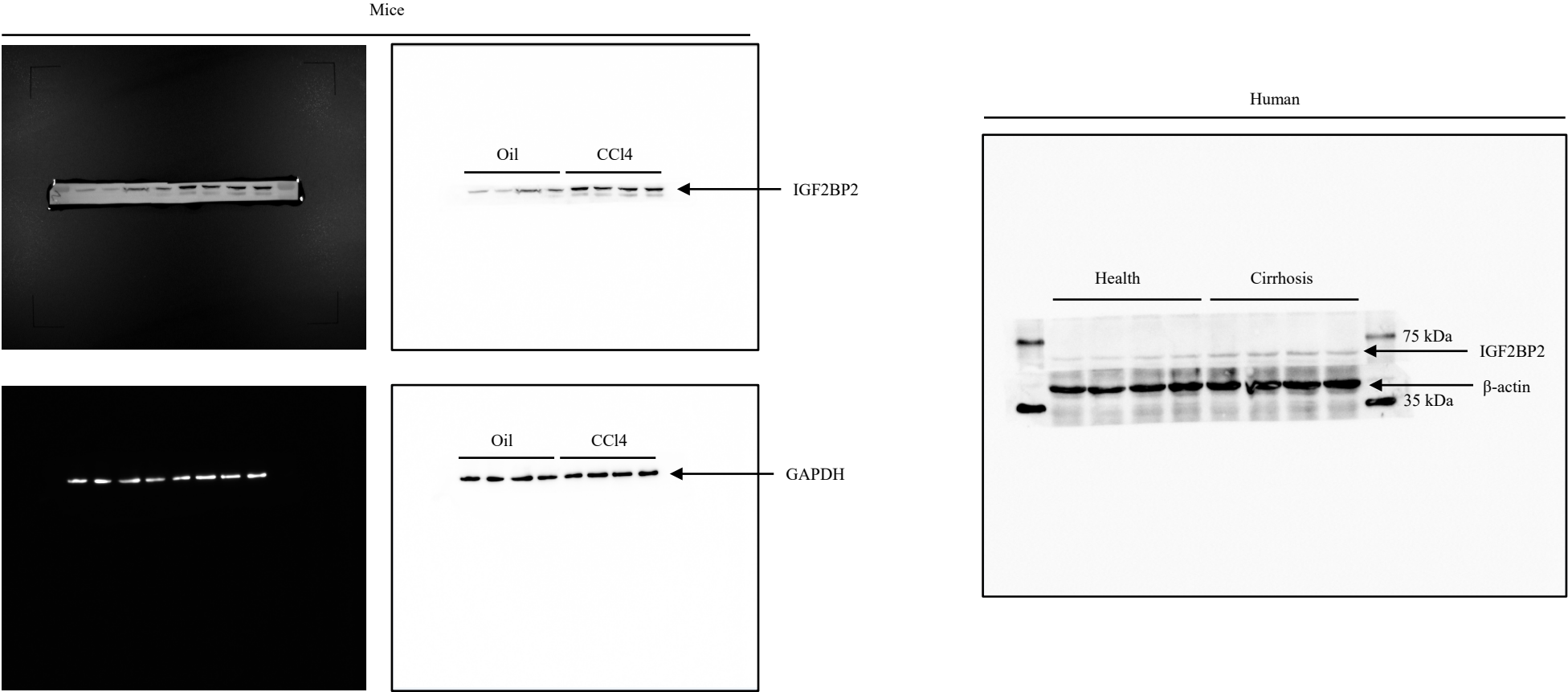

Figure S1E

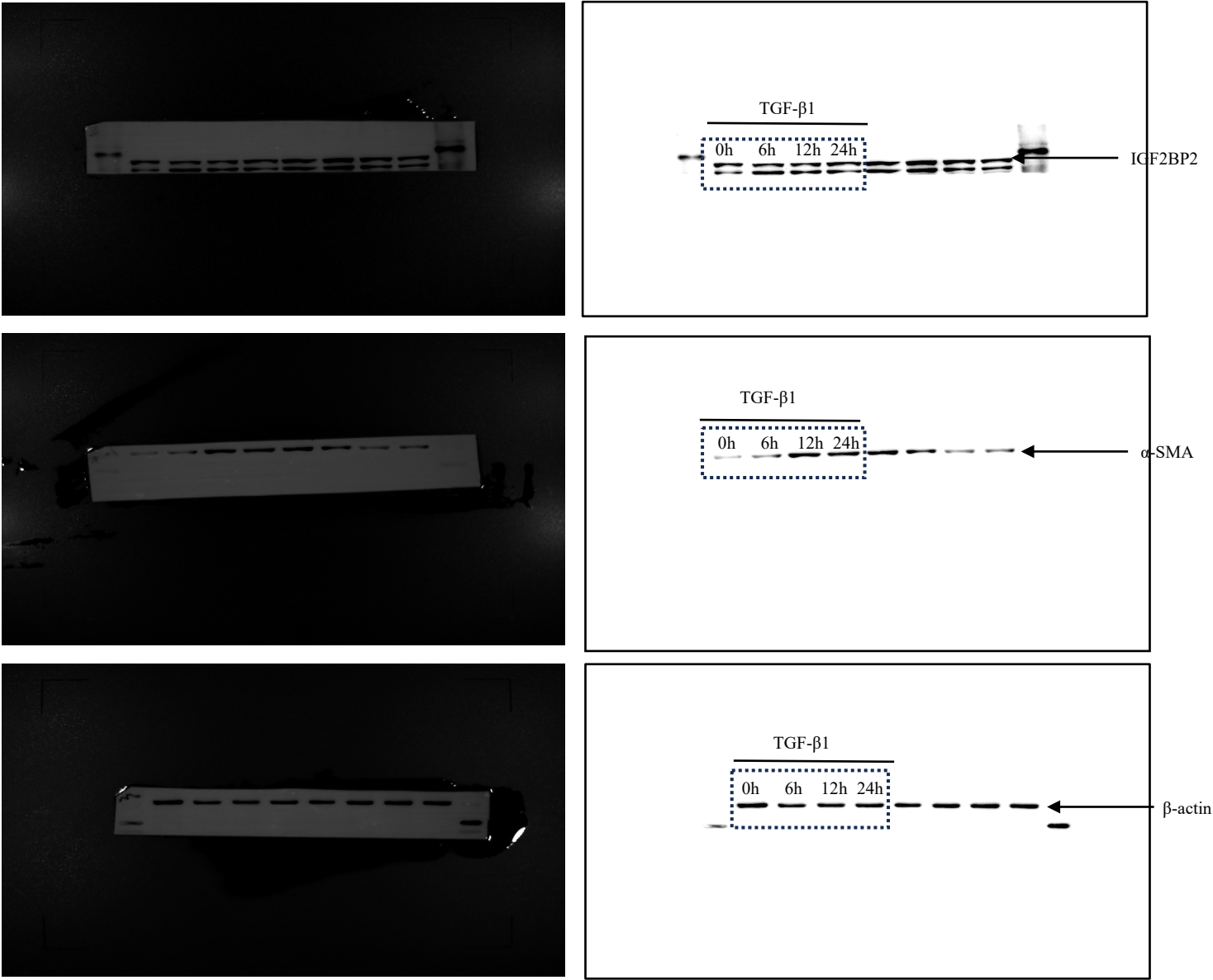

Figure 3B

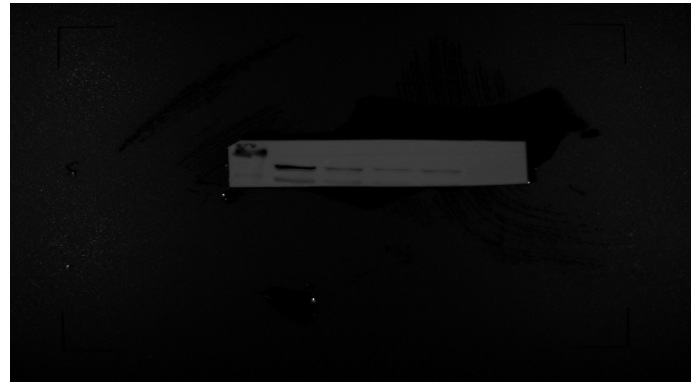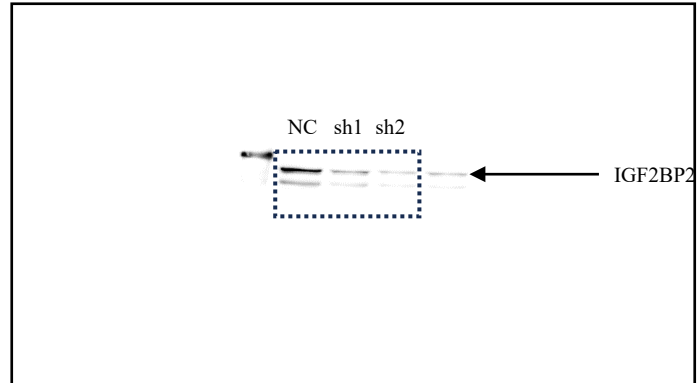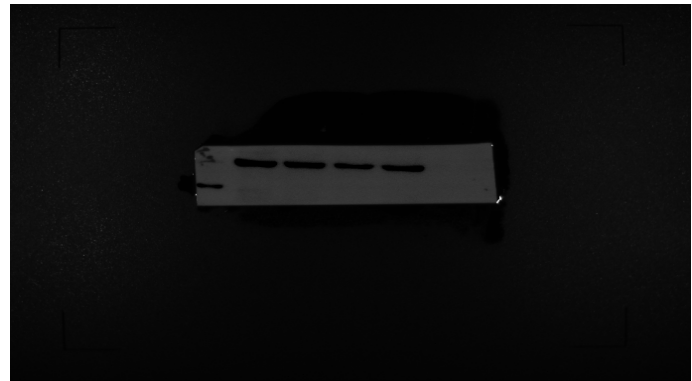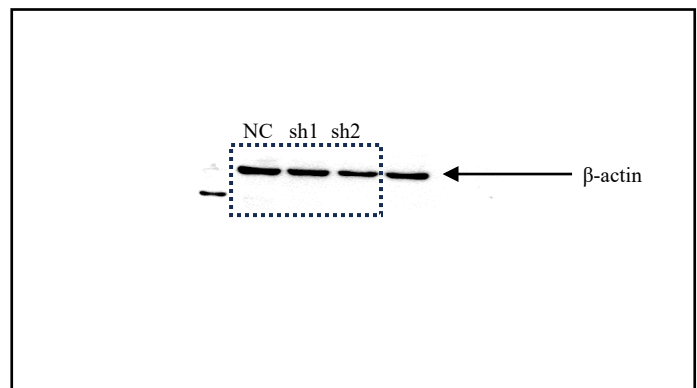

Figure 5O

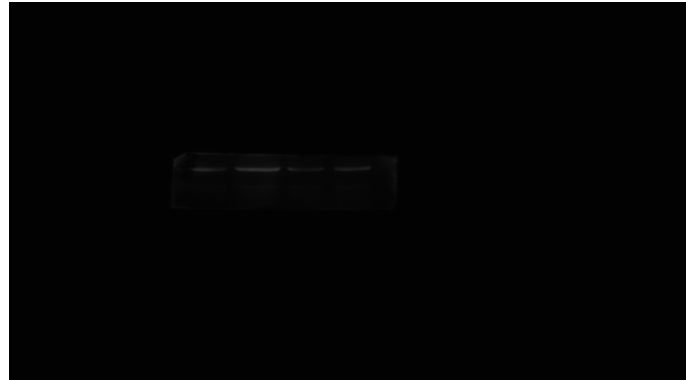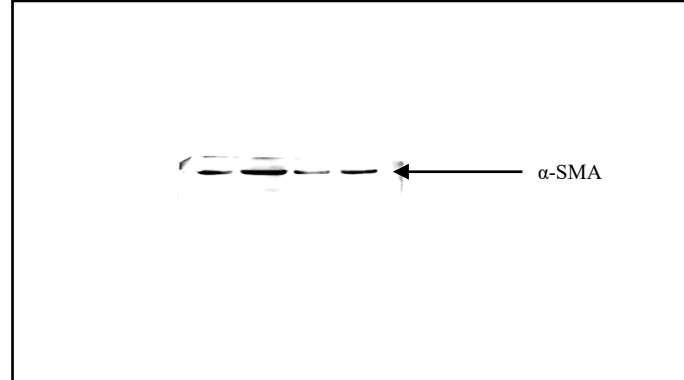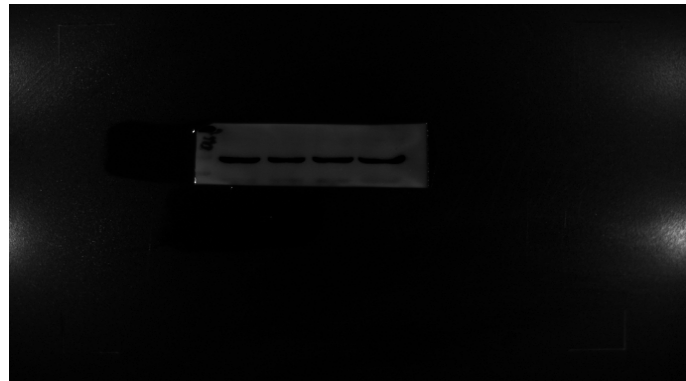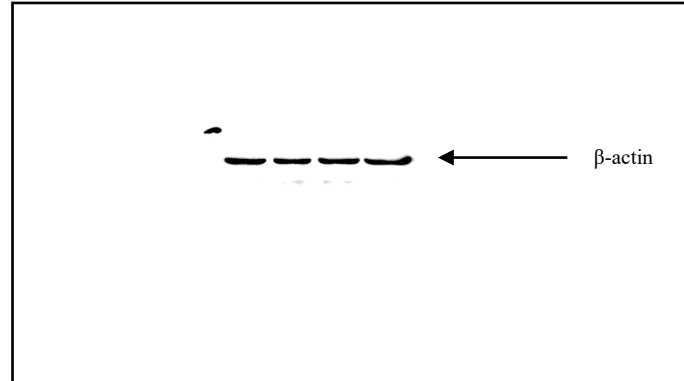

Figure 6G

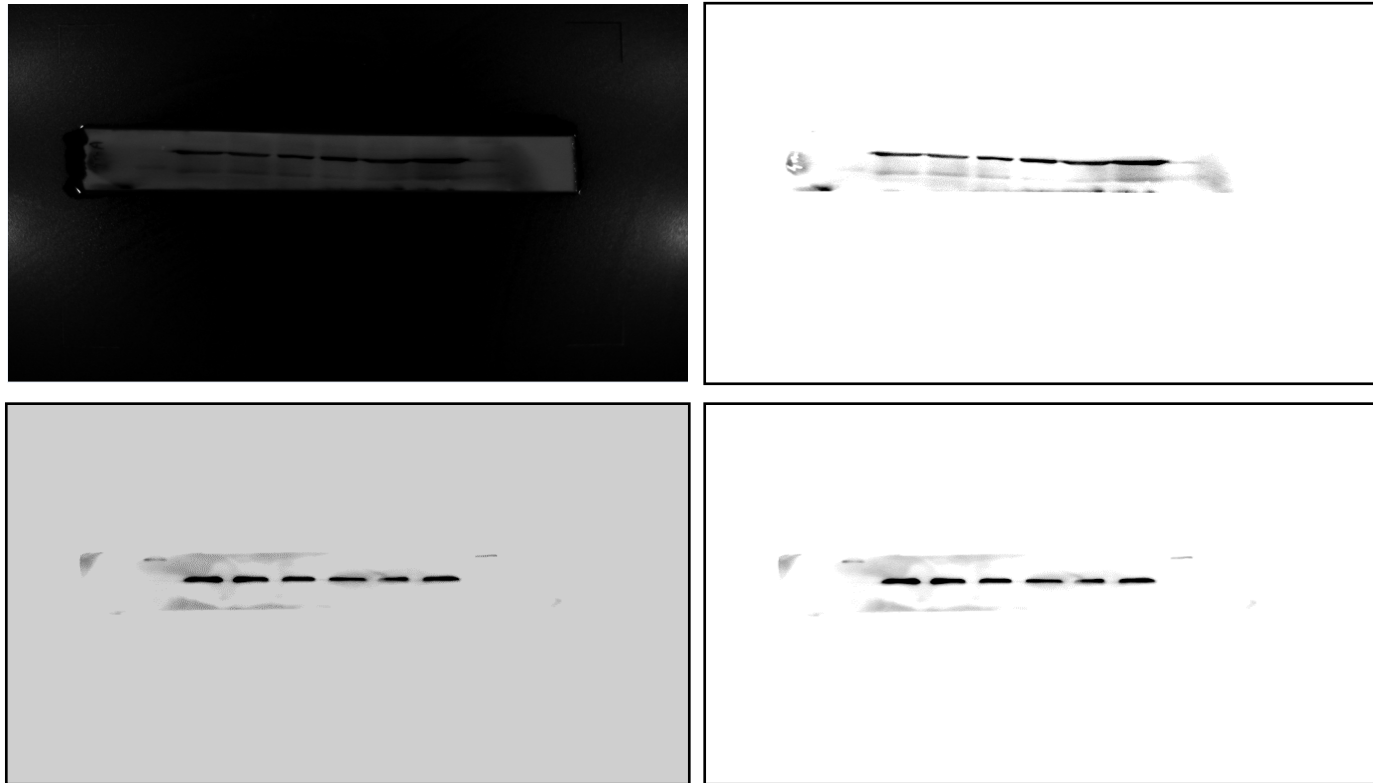

Figure 6K

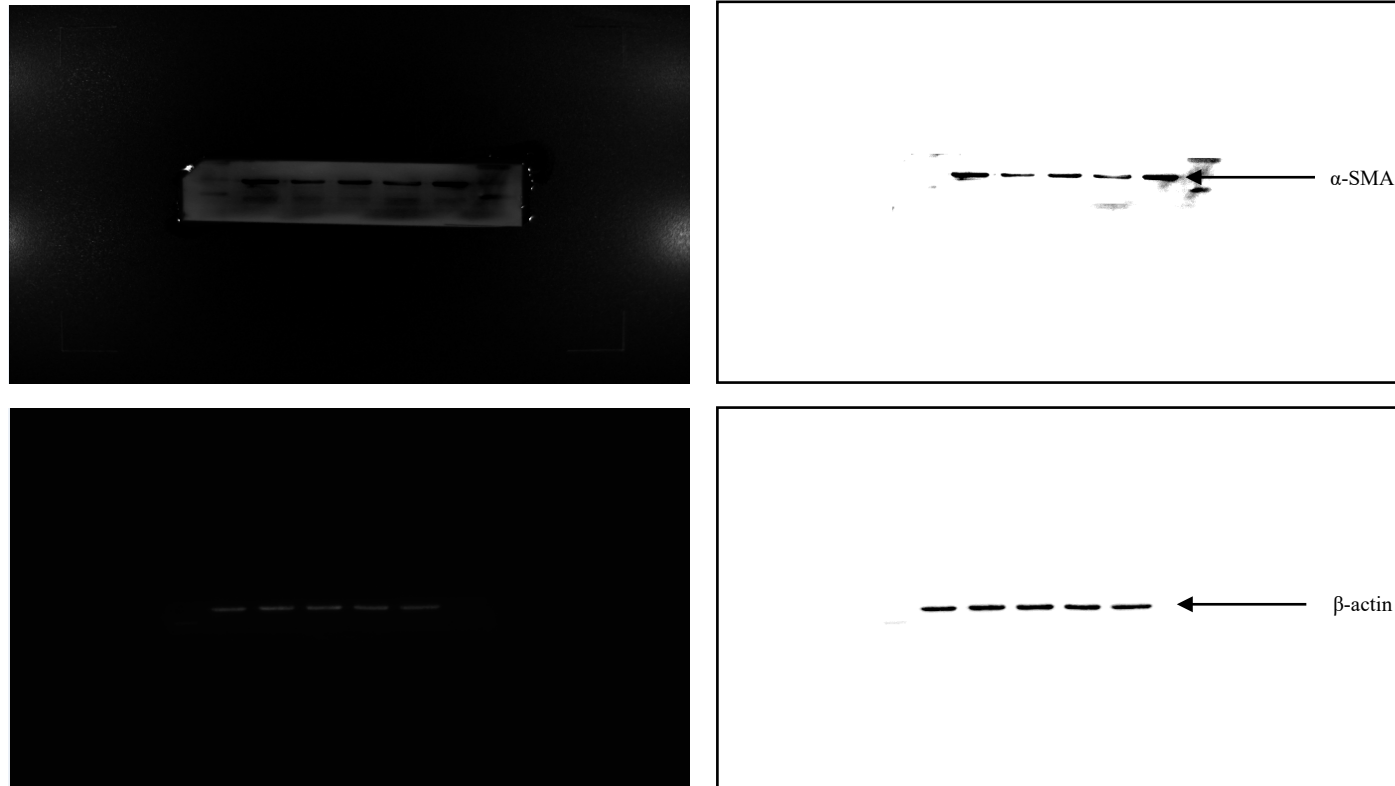

Supplement: Supplementary file 10 — Original Data File [file 41419_2024_6509_MOESM10_ESM.pdf]
