# Supplementary material for: The m6A reader IGF2BP2 regulates glycolytic metabolism and mediates histone lactylation to enhance hepatic stellate cell activation and liver fibrosis
Source: Cell Death Dis. 2024 Mar 5;15(3):189. doi: 10.1038/s41419-024-06509-9 (PMC10914723; doi:10.1038/s41419-024-06509-9)
Supplement: Supplementary file 5 — Supplementary Figure Legends [file 41419_2024_6509_MOESM5_ESM.pdf]

Figure S1. IGF2BP2 is upregulated in activated HSCs.

(A) The cell types in which IGF2BP2 is predominantly expressed in the liver were retrieved from the Human Protein Atlas (https://www.proteinatlas.org/). (B) *IGF2BP2* expression in quiescent HSCs and activated HSCs from GSE67664. (C) *IGF2BP2*, *ACTA2*, and *COL1α1* mRNA expression in LX-2 cells cultured in 2% FBS and 10% FBS for 1 h. (D) *IGF2BP2*, *ACTA2*, and *COL1α1* mRNA expression in LX-2 cells at different time points (0 h, 1 h, 6 h, 12 h) of TGF-β1 treatment. (E) Immunoblotting showing IGF2BP2 and α-SMA in LX-2 cells at different time points (0 h, 6 h, 12 h, 24 h) of TGF-β1 treatment. n.s., not significant; *$P < 0.05$, **$P < 0.01$, ***$P < 0.001$, ****$P < 0.0001$.

Figure S2. GO enrichment analyses in *IGF2BP2* KD LX-2 cells.

Bubble diagram showing GO enrichment analysis to demonstrate molecular function, cellular component, or biological processes in *IGF2BP2* KD LX-2 cells. The y-axis is for GO terms. The size and color of the bubbles represent the number and significance of genes associated with the term, respectively.

Figure S3. Inhibition of IGF2BP2 blocks lactate production in vitro.

(A) Measurement of lactate production in the serum of mice with CCl_4-induced liver fibrosis and healthy mice (n = 6). (B-C) Measurement of lactate levels (B) and glucose levels (C) in *IGF2BP2* KD LX-2 cells at 48 h. (D) Measurement of ATP content in *IGF2BP2* KD or TGF-β1-treated LX-2 cells at 24 h. (E-F) Measurement of lactate

levels (at 48 h) and ATP content (at 24 h) in LX-2 cells treated with TGF-β1 or JX5.

*P < 0.05, **P < 0.01, ***P < 0.001, ****P < 0.0001.
