# Supplementary material for: The m6A reader IGF2BP2 regulates glycolytic metabolism and mediates histone lactylation to enhance hepatic stellate cell activation and liver fibrosis
Source: Cell Death Dis. 2024 Mar 5;15(3):189. doi: 10.1038/s41419-024-06509-9 (PMC10914723; doi:10.1038/s41419-024-06509-9)
Supplement: Supplementary file 6 — Mouse AAV sequences and vector genomes (vg) [file 41419_2024_6509_MOESM6_ESM.doc]

**Table S1. Mouse AAV sequences and vector genomes (vg)**

| Target | Sequences | vector genomes/mouse/injection |
| --- | --- | --- |
| AAV-shRNA-1 | CCGTTGTCAACGTCACCTATA | 4 × 10^11^ vg/mL |
| AAV-shRNA-2 | CTGTACCCTCATCACCATTTC | 2 × 10^11^ vg/mL |
| AAV-NC | GAAGTCGTGAGAAGTAGAA | 2 × 10^11^ vg/mL |
