# Supplementary material for: The m6A reader IGF2BP2 regulates glycolytic metabolism and mediates histone lactylation to enhance hepatic stellate cell activation and liver fibrosis
Source: Cell Death Dis. 2024 Mar 5;15(3):189. doi: 10.1038/s41419-024-06509-9 (PMC10914723; doi:10.1038/s41419-024-06509-9)
Supplement: Supplementary file 7 — Sequence information for all RNA interference in this study [file 41419_2024_6509_MOESM7_ESM.doc]

**Table S2. Sequence information for all RNA interference in this study**

| Target | Sequences (5′-3′) |
| --- | --- |
| shIgf2bp2-1 | ctTAACCAGTGCAGAAGTCAT |
| shIgf2bp2-2 | cgGATCTTTGGGAAACTGAAA |
| shNC | TTCTCCGAACGTGTCACG |
| siLDHA | GCCGUCUUAAUUUGGUCCATT |
| siLDHB | GGGAGCUUAUUUCUUCAGATT |
