# Supplementary material for: The m6A reader IGF2BP2 regulates glycolytic metabolism and mediates histone lactylation to enhance hepatic stellate cell activation and liver fibrosis
Source: Cell Death Dis. 2024 Mar 5;15(3):189. doi: 10.1038/s41419-024-06509-9 (PMC10914723; doi:10.1038/s41419-024-06509-9)
Supplement: Supplementary file 8 — The specific primer sequences used in the qRT-PCR analysis in this study [file 41419_2024_6509_MOESM8_ESM.doc]

**Table S3. The specific primer sequences used in the qRT-PCR analysis in this study**

| Species | Target | Sequences (5′-3′) |
| --- | --- | --- |
| Human | *IGF2BP2* | Forward: CATCATCGGAAAGGAGGGCTTGAC |
|  |  | Reverse: GCATGGATGGTGACAGGCTTCTC |
|  | *ALDOA* | Forward: ACCTCAATGCCATTAACAAGTG |
|  |  | Reverse: TTAATAGGCGTGGTTAGAGACG |
|  | *COL1α1* | Forward: TGATCGTGGTGAGACTGGTCCTG |
|  |  | Reverse: CTTTATGCCTCTGTCGCCCTGTTC |
|  | *ACTA2* | Forward: CTTCGTTACTACTGCTGAGCGTGAG |
|  |  | Reverse: CCCATCAGGCAACTCGTAACTCTTC |
|  | *LDHB* | Forward: GAACTTGCTCTTGTGGATGTTT |
|  |  | Reverse: CTGAAGAAATAAGCTCCCATGC |
|  | *PGK1* | Forward: AGCCAAGTCGGTAGTCCTTATGAG |
|  |  | Reverse: ACAGTCCTTCAAGAACAGAACATCC |
|  | *ENO1* | Forward: TACCGCCACATCGCTGACTTG |
|  |  | Reverse: TGAGAACCGCCATTGATGACATTG |
|  | β-actin | Forward: GGCCAACCGCGAGAAGATGAC |
|  |  | Reverse: GGATAGCACAGCCTGGATAGCAAC |
| Mice | *Igf2bp2* | Forward: CGCCAGACGAGAATGAGGAAGTG |
|  |  | Reverse: GTATCTCTGCTCCTGCTGCTTCAC |
|  | *Col1α1* | Forward: GACAGGCGAACAAGGTGACAGAG |
|  |  | Reverse: CAGGAGAACCAGGAGAACCAGGAG |
|  | *Acta2* | Forward: CGTGGCTATTCCTTCGTGACTACTG |
|  |  | Reverse: CGTCAGGCAGTTCGTAGCTCTTC |
|  | β-actin | Forward: TATGCTCTCCCTCACGCCATCC |
|  |  | Reverse: GTCACGCACGATTTCCCTCTCAG |
