# Supplementary material for: The m6A reader IGF2BP2 regulates glycolytic metabolism and mediates histone lactylation to enhance hepatic stellate cell activation and liver fibrosis
Source: Cell Death Dis. 2024 Mar 5;15(3):189. doi: 10.1038/s41419-024-06509-9 (PMC10914723; doi:10.1038/s41419-024-06509-9)
Supplement: Supplementary file 9 — Primary antibodies used for immunoblotting [file 41419_2024_6509_MOESM9_ESM.doc]

**Table S4. Primary antibodies used for immunoblotting**

| Antibodies | Source | Identifier (Cat#) | Dilution |
| --- | --- | --- | --- |
| IGF2BP2 polyclonal antibody | Proteintech | 11601-1-AP | 1:2000 |
| ALDOA Polyclonal antibody | Proteintech | 11217-1-AP | 1:5000 |
| LDHA (C4B5) Rabbit mAb | Cell Signaling Technology | 3582 | 1:1000 |
| α-SMA Monoclonal Antibody | Signalway Antibody | 40482 | 1:5000 |
| LDHB Polyclonal Antibody | Elabscience | E-AB-40383 | 1:1000 |
| Anti-L-lactyl lysine rabbit pAb | PTM Biolabs | PTM-1401 | 1:1000 |
| L-Lactyl-Histone H3 (Lys18) Rabbit mAb | PTM Biolabs | PTM-1427RM | 1:1000 |
| Histone-H3 Polyclonal antibody | Proteintech | 17168-1-AP | 1:2000 |
| β-actin | Servicebio | GB15001-100 | 1:1000 |
| GAPDH | Cell Signaling Technology | 5174 | 1:1000 |
